# *RUNX1* mutations in blast-phase chronic myeloid leukemia associate with distinct phenotypes, transcriptional profiles, and drug responses

**DOI:** 10.1038/s41375-020-01011-5

**Published:** 2020-08-11

**Authors:** Shady Adnan Awad, Olli Dufva, Aleksandr Ianevski, Bishwa Ghimire, Jan Koski, Pilvi Maliniemi, Daniel Thomson, Andreas Schreiber, Caroline A. Heckman, Perttu Koskenvesa, Matti Korhonen, Kimmo Porkka, Susan Branford, Tero Aittokallio, Matti Kankainen, Satu Mustjoki

**Affiliations:** 1 Hematology Research Unit Helsinki, University of Helsinki and Helsinki University Hospital Comprehensive Cancer Center, Helsinki, Finland; 2 Translational Immunology Research Program and Department of Clinical Chemistry and Hematology, University of Helsinki, Helsinki, Finland; 3 Clinical Pathology Department, National Cancer Institute, Cairo University, Cairo, Egypt; 4 iCAN Digital Precision Cancer Medicine Flagship, Helsinki, Finland; 5 Institute for Molecular Medicine Finland (FIMM), Helsinki Institute of Life Science (HiLIFE), University of Helsinki, Helsinki, Finland; 6 Department of Computer Science, Helsinki Institute for Information Technology (HIIT), Aalto University, Espoo, Finland; 7 Finnish Red Cross Blood Service, Helsinki, Finland; 8 Department of Genetics and Molecular Pathology, Centre for Cancer Biology, SA Pathology, Adelaide, Australia; 9 Division of Health Sciences, School of Pharmacy and Medical Science, University of South Australia, Adelaide, Australia; 10 Australian Cancer Research Foundation Genomics Facility, Centre for Cancer Biology, SA Pathology, Adelaide, Australia; 11 School of Biological Sciences, University of Adelaide, Adelaide, Australia; 12 Department of Mathematics and Statistics, University of Turku, Turku, Finland

**Keywords:** Chronic myeloid leukaemia, Cancer genetics, Translational research

## Abstract

Blast-phase chronic myeloid leukemia (BP-CML) is associated with additional chromosomal aberrations, *RUNX1* mutations being one of the most common. Tyrosine kinase inhibitor therapy has only limited efficacy in BP-CML, and characterization of more defined molecular subtypes is warranted in order to design better treatment modalities for this poor prognosis patient group. Using whole-exome and RNA sequencing we demonstrate that *PHF6* and *BCORL1* mutations, *IKZF1* deletions, and AID/RAG-mediated rearrangements are enriched in *RUNX1*^mut^ BP-CML leading to typical mutational signature. On transcriptional level interferon and TNF signaling were deregulated in primary *RUNX1*^mut^ CML cells and stem cell and B-lymphoid factors upregulated giving a rise to distinct phenotype. This was accompanied with the sensitivity of *RUNX1*^mut^ blasts to CD19-CAR T cells in ex vivo assays. High-throughput drug sensitivity and resistance testing revealed leukemia cells from *RUNX1*^mut^ patients to be highly responsive for mTOR-, BCL2-, and VEGFR inhibitors and glucocorticoids. These findings were further investigated and confirmed in CRISPR/Cas9-edited homozygous *RUNX1*^*−/−*^ and heterozygous *RUNX1*^−/mut^ BCR-ABL positive cell lines. Overall, our study provides insights into the pathogenic role of *RUNX1* mutations and highlights personalized targeted therapy and CAR T-cell immunotherapy as potentially promising strategies for treating *RUNX1*^mut^ BP-CML patients.

## Introduction

*RUNX1*, also known as core binding factor subunit alpha (*CBFA2*), is a transcription factor (TF) and an essential component of the core binding factor complex that plays a key role in hematopoiesis [1]. Somatic and germline alterations involving *RUNX1* gene are commonly encountered in a variety of hematological malignancies [2]. *RUNX1* germline mutations are associated with familial platelet disorders (FPD) with predisposition to hematological malignancies [3]. In acute leukemia, *RUNX1* is affected by a range of chromosomal rearrangements resulting in fusions with multiple partners [4]. These include t(8;21) *RUNX1-RUNX1T1* translocation in 15% of AML patients [5], t(12;21) *ETV6-RUNX1* translocation in 25% of BCP-ALL patients [6], and t(3;21) *RUNX1-MECOM* in therapy-related MDS/AML patients [7]. In the t(12;21) *ETV6-RUNX1* ALL, it has been reported that the aberrant RAG recombination activity mediates off-target deletions and is the driver mutagenic mechanism [8]. In normal physiology, activation-induced cytidine deaminase (AID)/RAG axis is important in V(D)J rearrangement and somatic hypermutation (SHM) process during B lymphocyte development [9, 10].

Somatic *RUNX1* mutations are frequent among hematological malignancies like AML [11], ALL [12], MDS [13], and MDS/MPN (CMML) [14]. AML with mutated *RUNX1* (*RUNX1*^mut^ AML) is a provisional entity which accounts for 10% of the newly diagnosed patients and associates with an inferior prognosis [11, 15]. *RUNX1* mutations are frequently encountered in AML patients with minimal differentiation (AML-M0), where it demonstrates a unique molecular signature with upregulation of B-lymphoid genes [16]. Aberrant expression of the lymphoid marker CD19 is frequently observed in t*(8;21)-*AML [17], representing an interesting target for immunotherapy [18]. *RUNX1*^*mut*^ AML shows associations with mutations affecting spliceosome (*SRSF2* and *SF3B1*), epigenetic modifiers (*ASXL1* and *EZH2*), and *PHF6* and *BCOR* genes [19, 20]. Furthermore, *FLT3-ITD* and *MLL-PTD* frequently coexist with *RUNX1* mutations, while fusion genes and *NPM1* mutations are mutually exclusive with *RUNX1* mutations [21].

*RUNX1* mutations have also been found in CML patients and linked to disease progression and inferior treatment responses [22–24]. In our previous study, *RUNX1* mutations were identified as recurrent events in BP-CML (3 out of 20 patients) [25]. In concord, functional studies in mice have shown that *RUNX1* mutations can contribute to blast transformation of CML [26, 27]. Nevertheless, little is known about the role of *RUNX1* mutations in BP-CML. We therefore investigated the mutational profiles of RUNX1-mutated (*RUNX1*^mut^) and wild-type (*RUNX1*^wt^) BP-CML patients with whole-exome and RNA sequencing and integrated public genomic data of BP-CML patients to increase accuracy. This approach allowed us to enlighten a novel mutagenesis role of *RUNX1* mutations that is coupled with the activation of AID/RAG axis. Gene expression profiling demonstrated characteristic transcriptional programming in *RUNX1*^mut^ cases including upregulation of stem cell and B-lymphoid genes. Using drug sensitivity profiling of primary leukemia cells and CRISPR/Cas9 *RUNX1* gene-edited CML cell lines, we identified novel effective targeted therapies and CD19-CAR T cells as a promising immunotherapeutic option. Our data provide a comprehensive genomic and functional characterization of *RUNX1*^mut^ BP-CML.

## Materials and methods

### Patients

Clinical and hematological features of BP-CML patients are summarized in Supplementary Table 1. CML diagnosis and progression were defined according to World Health Organization criteria [28]. All subjects gave their written informed consent in accordance with the declaration of Helsinki. In addition, we integrated whole-exome and RNA-sequencing data from previously published BP-CML patients [24].

### Cell lines

Baf3 cells transfected with *P210-BCR-ABL1*-GFP were a gift from Prof. Nikolas von Bubnoff, Universitätsklinikum Freiburg, Germany. K562 was obtained from DSMZ (German Collection of Microorganisms and Cell Cultures). Both cell lines were cultured in RPMI-1640 (Lonza) supplemented with 10% FBS, 2-mM L-glutamine (Lonza), and 100-U/mL penicillin and 100-μg/mL streptomycin (Gibco).

### Flow cytometry analysis

Patient samples (bone marrow mononuclear cells (BMNCs)) and cell lines were stained with relevant panels of antibodies as indicated in the Supplementary materials, using recommended manufacturer protocols for surface antibodies staining. Cells were acquired with the FACS Verse and analyzed with FlowJo software (Version10.0.8r1, TreeStar). All antibodies were purchased from BD Biosciences, San Diego, CA, USA.

### Whole-exome sequencing (WES), RNA sequencing, and data analysis

Genomic DNA was extracted from BP-CML patients’ BMNCs. Skin samples were collected and used as germline controls to identify somatic mutations. WES protocol has been described in the earlier study [25]. The mean coverage depth was 138× (range: 99.9×−168.4×) (Supplementary Table 1). Regarding RNA sequencing, RNA isolation and RNA-sequencing protocol have been described earlier [25]. Details of RNA-sequencing workflow, bioinformatics analysis, and adjustment for possible confounding factors are described in Supplementary materials.

### Drug sensitivity and resistance testing (DSRT)

The oncology compounds library, employed to test patient samples, consisted of 125 FDA/EMA anticancer approved drugs and 127 investigational and preclinical compounds. For cell lines, a comprehensive library of 528 compounds (156 approved drugs and 372 investigational compounds) was used. Drugs were tested in five increasing concentrations over a 10,000-fold range. For drug combination testing, the selected drugs were tested with dose–response matrices comprising seven different concentrations. DSRT was performed as previously described [29], and quantification of DSS and drug synergy scores is described in Supplementary materials.

### CRISPR/Cas9 RUNX1 gene editing

Baf3-*BCR-ABL1* cells were transfected with pU6-(BbsI)-CBh-Cas9-T2A-mCherry (Addgene plasmid#64324) expressing CRISPR-Cas9 and sgRNA targeting exon-4 of *runx1* gene using Fugene HD transfection reagent (Promega). All of sgRNA, primers, oligos, plasmids, and antibodies are listed in Supplementary Table 2. Selection of clones and validation of editing is described in Supplementary materials.

### Generation of and phenotyping of CAR T cells and ex vivo CAR T cells cytotoxicity assay

CAR T cells were manufactured and the ex vivo cytotoxicity assay was performed as previously described [30, 31] and indicated in the Supplementary methods. The cells were stained using a designed antibodies panel (Supplementary Table 3). Cells were acquired using iQue Screener Plus flow cytometer and analyzed using the ForeCyt software (edition 6.2, Intellicyt). Details of data analysis can be found in Supplementary materials.

### Statistical analysis

Two-tailed Student *t* test, Mann–Whitney *U*-test, Fisher Exact test, Spearman correlation, Pearson’ correlation tests, and Fisher’s Exact Test with simulated *p* value on 1*e* + 07 replicates were computed using GraphPad Prism 7 software or R 3.5.0.

## Results

### RUNX1 mutations are frequent in BP-CML and co-occur with IKZF1 deletions and PHF6 and BCORL1 mutations

**Fig. 1:**
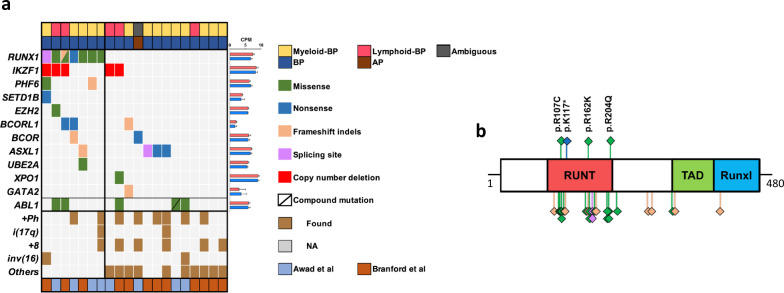
Spectrum of *RUNX1* mutations in BP-CML patients. **a** Landscape of somatic mutations identified by WES in BP-CML samples from our cohort (*n* = 8, 4 *RUNX1*^mut^ and 4 *RUNX1*^wt^) and from Branford et al. [24] (*n* = 12, 3 *RUNX1*^mut^ and 9 *RUNX1*^wt^). Complete lists of identified mutations are detailed in Supplementary Table 4. Explanatory tracks from top to bottom show phenotype of the blast (myeloid-BP, lymphoid-BP, and Ambiguous) and phase of CML (accelerated phase AP or blast phase BP) cases. The filling color indicates the type of the variant. The average expression of the genes in 4 *RUNX1*^mut^ (red) and 5 *RUNX1*^wt^ (blue) BP-CML samples is shown on the right expressed as counts per million mapped reads (CPMs). Bar lengths indicate means and errors. Chromosomal abnormalities, including recurrent abnormalities and high-risk leukemia-associated abnormalities, are shown in the lower part of the plot. The bottom explanatory track indicates the study cohort. **b** Schematic diagram of the protein structure and domains of *RUNX1* protein and position of mutations in BP-CML. RUNT domain (85–206), TAD (318–389), and RunxI (389–480). Each diamond represents one call of the variant and the fill color represents the type/predicted change of the variants. Diamonds in the upper panel represent variants detected in this study and diamonds in the lower panel represent *RUNX1* variants previously called in published BP-CML data [22, 24] (see also Supplementary Fig. 1a).

We analyzed thirteen samples from eight BP-CML patients with a median age of 45 years (range 24–74 years) using WES (Supplementary Table 1). Serial samples were available for four patients either from diagnostic CP-CML (*n* = 1), relapse (*n* = 2), or both (*n* = 1). In our WES cohort (marked with Awad et al. in the Fig. 1a and Supplementary Fig. 1a), four patients harbored somatic mutations in the *RUNX1* gene. *RUNX1* mutations included three missense mutations (p.R162K, p.R204Q, and p.R107C) and one nonsense mutation (p.K117*), that were all located in the Runt domain (Fig. 1b). Myeloid BP and lymphoid BP were nearly evenly distributed between *RUNX1*^mut^ and *RUNX1*^wt^ groups (*p* > 0.99, Fig. 1a). Blasts from myeloid-BP *RUNX1*^mut^ patients frequently expressed HLA-DR, TdT, and aberrant lymphoid markers (CD19 or CD7 in two out three myeloid *RUNX1*^mut^ BP patients) (Supplementary Table 1). *RUNX1*^mut^ BP-CML patients showed a notable population of plasmacytoid dendritic cells (pDCs) in contrast to *RUNX1*^wt^ group (Supplementary Fig. 1b).

To enable comprehensive profiling of the mutational landscape of *RUNX1*^mut^ BP-CML patients, we reanalyzed WES data from Branford et al. publication [24]. We recovered four *RUNX1* mutations (p.T176fs, p.L175Q, p.D198G, c.508+2T>C splice donor) in three patients (Supplementary Table 4). We also supplemented the data with Grossman et al. publication [22] in which targeted sequencing approach had been used. Frequent co-occuring mutations in *RUNX1*^mut^ patients included *PHF6* and *BCORL1* mutations (Fig. 1a, Supplementary Fig. 1a, and Supplementary Table 4). *IKZF1* deletions were more common in *RUNX1*^mut^ patients, but also found in lymphoid *RUNX1*^wt^ BP-CML patients (Fig. 1a). In mut2 patient with longitudinal samples, a *RUNX1* mutation (p.R162K) was seen also in diagnosis (CP) sample (variant allele frequency, VAF = 58%), with acquisition of loss of heterozygosity and loss of the wild-type allele prior sampling at BP (VAF = 99%) (Supplementary Fig. 1c).

### *RUNX1* mutations confer a distinct mutational signature with characteristic AID/RAG-mediated activity

**Fig. 2:**
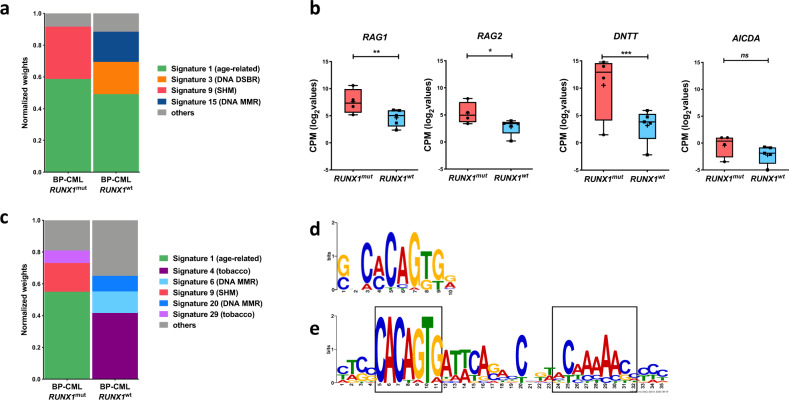
Cancer signatures and mutation loads of *RUNX1*^mut^ patients highlight the contribution of AID/RAG pathway to mutagenesis. **a** Normalized weights of trinucleotide signatures in four *RUNX1*^mut^ BP and four *RUNX1*^wt^ patients highlighted the major contribution of signature-9 (related to AID/RAG pathway) in *RUNX1*^mut^ BP patients. Weights of the three most frequent signatures (if applicable) in each cancer type are shown across cancers as separate signatures. Total weight of all other signatures is shown under the category “other.” **b** Expression levels (CPM log_2_ values) of *RAG1, RAG2*, and *DNTT* genes are significantly higher in *RUNX1*^mut^ patients compared with *RUNX1*^wt^ patients (*p < 0.05, **p < 0.01, ***p < 0.005, two-tailed student’s test). **c** Normalized weights of trinucleotide signatures from combined data including 7 *RUNX1*^mut^ and 13 *RUNX1*^wt^ BP-CML patients underscored the association of signature-9 with *RUNX1*^mut^ in BP-CML patients. Weights of the three most frequent signatures in each cancer type are shown across cancers as separate signatures. Total weight of all other signatures is shown under the category “other.” **d** RAG-RSS heptamer sequence identified by agnostic motif search using MEME in 23/32 breakpoints in *RUNX1*^mut^ BP (*E* value = 1.7 × 10^–14^) and in 16/39 breakpoints in *RUNX1*^wt^ patients (*E* value = 1.4 × 10^–14^) within 20 bp of breakpoint junctions. **e** RAG canonical RSS, heptamer, and nanomer sequences (in boxes) separated by 12-bp spacer, identified by agnostic motif search using MEME in 16/32 breakpoints in *RUNX1*^mut^ BP within 100 bp of breakpoint junctions (*E* value = 8.0 × 10^–46^).

To elucidate the active mutational processes in BP-CML patients, we performed mutational signature analysis of the called variants. Age-related signature, DNA double-strand break repair, and DNA mismatch repair signatures revealed notable contribution to the mutational profile of BP-CML patients (Fig. 2a). Signature-9 was prominent in *RUNX1*^mut^ samples, including myeloid-BP *RUNX1*^mut^, but absent in *RUNX1*^wt^ samples. Signature-9 is related to AID/RAG activity and polymerase *η*-induced SHM [32]. Notably, several AID/RAG components, including *RAG1, RAG2, AICDA,* and *DNTT* genes, were overexpressed in *RUNX1*^mut^ compared with *RUNX1*^wt^ patients (Fig. 2b). Extension of the analysis to the combined data of 20 BP-CML patients (*RUNX1*^mut^; *n* = 7, *RUNX1*^wt^; *n* = 13) showed no significant differences in the mutational load or structural variants (SV) between *RUNX1*^mut^ and *RUNX1*^wt^ patient samples (Supplementary Fig. 2a–c and Supplementary Table 5). Mutational signature profiles of *RUNX1*^mut^ patients from both cohorts showed significant similarity (Supplementary Fig. 2d and Supplementary Table 6), and SHM signature-9 demonstrated enrichment in *RUNX1*^mut^ patients’ profile exclusively also in the combined dataset (Fig. 2c).

Given the enrichment of SHM signature-9 in mutation profiles of *RUNX1*^mut^ cases, an unsupervised motif search algorithm was used to explore contribution of AID/RAG-mediated recombination events to SV events. We first analyzed the 20-bp sequence spanning the breakpoint. The perfect heptamer sequence CACAGTG was significantly enriched in *RUNX1*^mut^ patients compared with *RUNX1*^wt^ group (*p* < 0.01). RAG heptamer was demonstrated in 31 sites involving one or both ends of 23/32 (71.9%) of breakpoints in *RUNX1*^mut^ patients (*E* value = 1.7 × 10^–14^) compared with 20 sites involving 16/39 (41%) of breakpoints in *RUNX1*^wt^ patients (Fig. 2e). By increasing the size of the output motif, the RAG canonical RSS motif (conserved heptamer (CACAGTG) and nonamer (ACAAAAACC) separated by a 12-bp spacer) was only enriched around breakpoints in *RUNX1*^mut^ patients (16 sites involving 12/32 (37.5%) of breakpoints, *E* value = 8.0 × 10^–46^) (Fig. 2f and Supplementary Fig. 3). Interestingly, we observed RAG-RSS at both ends of an intragenic *IKZF1* deletion in a *RUNX1*^mut^ patient.

### *RUNX1* mutations induce upregulation of stem cell and B-lymphoid markers, interferon signaling, and immune-related pathways

**Fig. 3:**
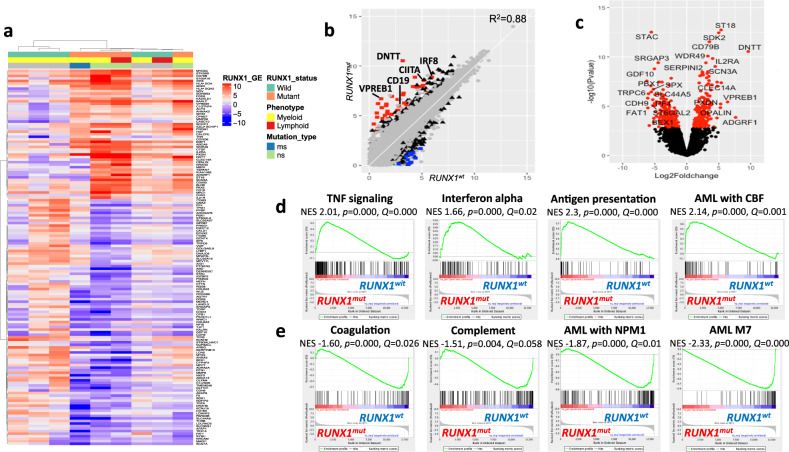
Transcriptional profiling of BP-CML patients demonstrate upregulation of stem cell and lymphoid markers, interferon signaling, and dysregulation of immune-related pathways in *RUNX1*^mut^ BP-CML patients. **a** Heatmap of top statistically differentially expressed genes (*Q* < 0.05, two-tailed student’s test) correlating with *RUNX1* mutations with absolute logFC > 3. Fading blue colors indicate downregulation of the gene in the sample and red its upregulation relative to the mean expression of the genes across all samples. Explanatory tracks from top to bottom show *RUNX1* status, blast phenotype, and mutation type. Clustering was performed for both genes and samples using the Euclidean distance and Ward linkage method. **b** Correlation of expression levels of all protein-coding genes between *RUNX1*^mut^ and *RUNX1*^wt^ subsets. Each gene is represented by a gray dot. Significantly differentially expressed genes (Bayesian statistical test, *Q* < 0.05) are represented by black triangles. Red and blue squares represent the top 50 upregulated and downregulated genes, respectively (Pearson correlation *R*^2^ = 0.88). **c** Volcano plot of protein-coding genes between *RUNX1*^mut^ (right) and *RUNX1*^wt^ (left). Each gene is represented by a black dot, and significant differentially expressed genes (*Q* < 0.05, Bayesian statistical test) are colored red. **d** GSEA of TNF, IFN-alpha, IFN-gamma, and CBF-AML pathways upregulated in *RUNX1*^mut^ compared with *RUNX1*^wt^ patients. **e** GSEA of coagulation, complement, *NPM1*^mut^-AML, and AML-FAB M7 pathways downregulated in *RUNX1*^mut^ compared with *RUNX1*^wt^ patients.

To gain insights into the transcriptional changes induced by *RUNX1* mutations, diagnostic samples from four *RUNX1*^mut^ and five *RUNX1*^wt^ patients, were analyzed using RNA-sequencing (Fig. 3a). After adjusting for possible confounding factors, we identified 396 genes that were differentially expressed between *RUNX1*^mut^ and *RUNX1*^wt^ patients (*Q* < 0.05, Supplementary Table 7). Distinct phenotypic markers and TFs, including genes associated with hematopoietic stem cells (HSC) (*CD133/PROM1*, *BAALC, CD34*) and lymphoid progenitors (*DNTT, VPREB1, PAX5, CD19*) were upregulated, whereas markers of megakaryopoiesis, erythropoiesis, and granulopoiesis (*ITG3B/CD61, PF4, ABO*) were downregulated in *RUNX1*^mut^ patients (Fig. 3a–c and Supplementary Fig. 4). pDCs-specific TFs, including *IRF8* and *TCF4*, were similarly upregulated in *RUNX1*^mut^ patients. *RUNX1* mutations were associated with dysregulation of several immune regulatory molecules, including *CIITA, CD74, B7-H6 (NCR3LG1), CD69*, and multiple *HLA-DR* and *TLR* molecules, in addition to cytokine receptors (*IL2RA, IL21R, and IL12RB2*) (Fig. 3a–c and Supplementary Fig. 4).

Results from gene set enrichment analysis (GSEA) showed upregulation of interferon alpha and gamma signaling, antigen processing and presentation, TNF and MAPK signaling pathways in *RUNX1*^mut^ patients, whereas coagulation and complement pathways were the most downregulated (Fig. 3d, e). *RUNX1*^mut^ upregulated gene sets were enriched for HSC-specific pathways while differentiation-related (neutrophil-related) pathways were enriched in the downregulated sets. Expression profile of *RUNX1*^mut^ BP-CML patients shared similarities with CBF-related AML in contrast with *NPM1*^mut^-AML and AML with granulocytic or megakaryocytic differentiation (Supplementary Table 7).

Since our cohort had a dominance of myeloid-BP phenotype (6/9 patients), we investigated whether *RUNX1*^mut^-induced transcriptional changes can also be demonstrated in lymphoid-BP phenotype. We analyzed data of lymphoid-BP patients from Branford et al. [24] (*n* = 16 patients, *RUNX1*^mut^ = 7, *RUNX1*^wt^ = 9). Lymphoid-BP samples showed clustering according to *RUNX1* mutation status. Upregulation of several genes, including *BAALC, CD133, ST18,* and *FLT4*, was comparable to *RUNX1*^mut^ myeloid-BP profiles. Furthermore, GSEA demonstrated similarities of upregulated pathways between *RUNX1*^mut^ lymphoid-BP and CBF-related AML in contrast to *NPM1*^mut^-AML, highlighting *RUNX1*^mut^-specific transcriptional signature (Supplementary Fig. 5).

### *RUNX1*^mut^ BP-CML cells showed increased sensitivities to mTOR, VEGFR, BCL2 inhibitors, and glucocorticoids

**Fig. 4:**
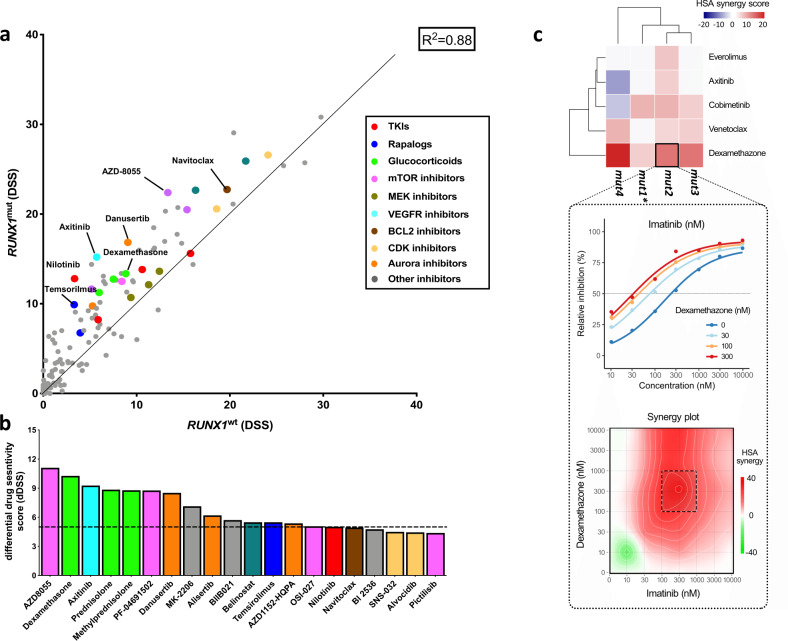
Drug response pattern characteristic of *RUNX1*^mut^ BP-CML patients. **a** Scatter plot comparing drug sensitivity score (DSS) of *RUNX1*^mut^ (*n* = 4) and *RUNX1*^wt^ BP-CML patients (*n* = 4). Color indicates different drug families (primary targets). **b** The top 20 targeted compounds with selective activity across *RUNX1*^mut^ BP-CML patient compared with *RUNX1*^wt^ BP-CML patient samples ranked by the difference of the median DSS scores between *RUNX1*^mut^ and *RUNX1*^wt^ groups, i.e., differential drug sensitivity score (dDSS). Conventional chemotherapeutic drugs (Supplementary Table 8) and broadly active compounds (CUDC-907, KX2–391, UCN-01, ONX-0914) are excluded. **c** Heatmap showing the highest single agent (HSA) synergy score when combining imatinib with each of the selected drugs (everolimus, axitinib, cobimetinib, venetoclax, dexamethasone) in four *RUNX1*^mut^ BP-CML patients (top panel). An example is highlighted that shows an increased potency of imatinib (decreased IC_50_) with increased dexamethasone concentrations (middle panel) and the corresponding HSA synergy plot of the imatinib-dexamethasone combination (bottom panel), showing synergy distribution and the most synergetic concentration window (dotted area). Asterisk indicates mut1 patient carrying gatekeeper *ABL1*-T315I resistance mutation. A full set of the synergy plots for all the combinations can be found in Supplementary Fig. 7.

Next, we explored how the *RUNX1* mutation-induced genomic changes modulate the drug responses of BP-CML cells. DSS were quantified for a panel of 255 oncology drugs using cells from eight BP-CML patients (Supplementary Table 8). Compared with *RUNX1*^wt^, *RUNX1*^mut^ patients showed greater sensitivity to mTOR inhibitors, VEGFR inhibitors, glucocorticoids, and navitoclax (Fig. 4a,b and Supplementary Fig. 6a). This selective activity was more notable when limiting the comparison to patients with myeloid-BP CML (Supplementary Fig. 6b, c). Overexpression of genes encoding targets for some of the identified drugs was observed in the *RUNX1*^mut^-associated transcriptional data, including *NR3C1* gene (glucocorticoid receptor) and *FLT4* gene (VEGFR3 receptor) (Supplementary Fig. 6d). Interestingly, cells from a patient with nonsense *RUNX1* mutation demonstrated enhanced sensitivity to glucocorticoids and mTOR inhibitors and reduced sensitivity to navitoclax, compared with those with *RUNX1* missense mutations (two patients) (Supplementary Fig. 6e).

Given the ex vivo effectiveness of the selected drugs, we tested whether the combination of these drugs with a TKI would enhance killing of *RUNX1*^mut^ blasts in the ex vivo setting. We tested cells from the *RUNX1*^mut^ (*n* = 4) and *RUNX1*^wt^ (*n* = 2) patients with imatinib in combination with dexamethasone, everolimus, cobimetinib, axitinib as well as venetoclax in a dose-dependent manner to investigate potential synergistic drug activities (Supplementary Fig. 7 and Supplementary Table 8). One patient carried gatekeeper *ABL1*-T315I resistance mutation, hence imatinib was not active and no synergy was detected (Fig. 4c). In *RUNX1*^mut^ patients, we were able to identify specific potential synergistic effects of imatinib-dexamethasone combination and to a lesser degree, imatinib-cobimetinib and imatinib-venetoclax combinations (Supplementary Fig. 7).

### In CML cell lines, *RUNX1* mutations induce phenotypic, transcriptional, and drug sensitivity profiles similar to *RUNX1*^mut^ BP-CML patients

**Fig. 5:**
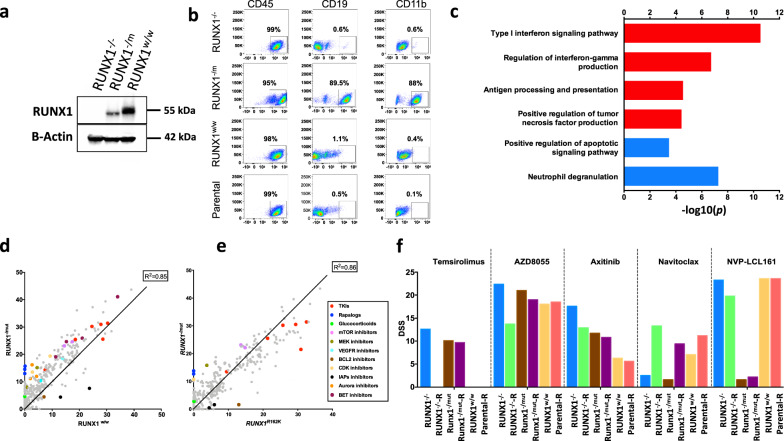
*RUNX1* CRISPR-edited CML cell lines show transcriptional reprogramming and DSRT profiles similar to changes in *RUNX1*-mut BP-CML patients. **a** Western plot of RUNX1 protein confirm efficient CRISPR-editing where *RUNX1*^−/−^ cell line shows complete loss of RUNX1 protein and *RUNX1*^−/mut^ cell line reduction of RUNX1 protein level compared with control. **b** Flow cytometry plot of CRISPR-edited and control cell lines. *RUNX1*^−/*mut*^ cell line shows induced phenotypic changes with expression of CD19 and CD11b in contrast to *RUNX1*^−^^*/−*^ and control lines. **c** Depiction of molecular pathways with significant altered expression between *RUNX1*^−/mut^ and *RUNX1*^wt/wt^ cell lines using the top differentially expressed genes with >2 log foldchange (the top 300 upregulated and the top 300 downregulated genes). The red bars are upregulated pathways and blue bars downregulated pathways. The analysis highlighted the reprogramming of expression of genes similar to *RUNX1*^*-*^^mut^ BP-CML patients’ profiles related to IFN, TNF, and antigen presentation pathways. Full lists of differentially expressed genes and enriched pathways are listed in Supplementary Table 9. **d** Correlation of DSS scores between *RUNX1*^−/mut^ and *RUNX1*^wt/wt^ cell lines, highlighting acquired sensitivity to (AZD8055, temsirolimus), MEK- (gedatolisib, cobimetinib), CDK- (SNS-032, AT7519), BET- (JQ1, birabresib), and VEGFR- (axitinib) inhibitors and resistance to XIAP inhibitors (NVP-LCL-161, AT-406). **e** Correlation of DSS scores between *RUNX1*^*R162K*^ (representing RUNX1 missense mutation) and *RUNX1*^−^^/mut^ (representing RUNX1 nonsense mutation) cell lines, highlighting differential activity of mTOR-, MEK- inhibitors (more active in nonsense mutated line), and BCL2 inhibitor (navitoclax is more active in missense mutated cell lines), similar to patient-derived primary cells. **f** Comparison of drug responses of Baf3-BCR-ABL1 CRISPR-edited (*RUNX1*^−/−^*, RUNX1*^−/mut^*, RUNX1*^wt/wt^) and parental cell lines to selected active agents temsirolimus, AZD8055 (mTOR inhibitors), axitinib (VEGFR inhibitor), navitoclax (BCL2 inhibitor), and NVP-LCL-161 (XIAP inhibitor). The bar height represents DSS scores. R indicates induced re-expression of wild-type *RUNX1* gene. Acquired drug activities in *RUNX1*^−^^*/−*^ line were lost with *RUNX1* re-expression (e.g., mTOR and VEGFR inhibitor differential activities), but not in *RUNX1*^−/mut^ cell line where the mutant *RUNX1* is driving the drug sensitivities (e.g., NVP-LCL-161 resistance).

Given the complex genetic background of BP-CML patients, we next validated whether the identified transcriptional and drug sensitivity characteristics are truly specific to *RUNX1* mutations. We used a mouse Ba/f3 cell line transduced with *P210-BCR-ABL1* expression vector as a model of CP-CML to simulate the impact of *RUNX1* mutations on the disease phenotype. We created a *RUNX1*^−^^*/−*^ cell line with complete *RUNX1* knockdown (homozygous deletion) and a *RUNX1*^−/mut^ cell line with an in-frame deletion (−3), predicted to have a deleterious effect on protein function, together with an out of frame (−1) deletion (heterozygous deletion) using CRISPR-cas9 technology, that was validated using western blot of RUNX1 protein (Fig. 5a and Supplementary Fig. 8a,b). Phenotypic analysis showed an induced expression of CD19 in *RUNX1*^−^^/mut^ cell line, but neither in *RUNX1*^−/−^ line, wild-type control line (*RUNX1*^wt/wt^*)* nor parental cell line (Fig. 5b). RNA sequencing of the CRISPR-edited cell lines demonstrated enrichment of *RUNX1* target genes and *RUNX1*-related pathways in the downregulated gene sets in *RUNX1*^*−/−*^ line, compared with wild-type control line. The transcriptional profile of the *RUNX1*^−/mut^ cell line shared many similarities with *RUNX1*^mut^ BP-CML patient profiles (Supplementary Table 9). Interferon signaling, inflammatory response, and antigen presentation pathways were upregulated while neutrophil degranulation and differentiation pathways were downregulated (Fig. 5c).

Next, we compared the drug sensitivity profiles of *RUNX1*-edited cell lines. *RUNX1*^−/mut^ showed higher sensitivity to mTOR-, VEGFR- and CDK- inhibitors in agreement with patient DSRT profiles. In addition, *RUNX1*^−^^/mut^ demonstrated selective resistance to IAPs inhibitors (NVP-LCL-161, birinapant, and AT-406) and BET inhibitors (JQ, birabresib, and I-BET151), which were not tested in the patient samples (Fig. 5d,e and Supplementary Table 10). In addition to Ba/f3 cell line, we also created a K562-*RUNX1*^*−/−*^ cell line, which showed similar drug sensitivity profile (Supplementary Fig. 8c). Induced re-expression of wild-type *RUNX1* gene was able to restore the sensitivity patterns of the parental cell line to the selected compounds in *RUNX1*^*−/−*^ but not in *RUNX1*^−/mut^ cells, confirming specificity of the induced DSRT changes to the *RUNX1* status (Fig. 5f). Furthermore, introduction of *RUNX1 p.R162K* mutation in Baf3-*BCR-ABL1* or K562 cell lines induced changes in the sensitivity profiles, including enhanced activity of navitoclax, AZD8055, and axitinib similar to *RUNX1*^mut^ patients’ profiles (Supplementary Fig. 8d,e). Interestingly, differential drug activity associations with somatic mutation types (e.g., enhanced mTOR activity with nonsense mutations and navitoclax activity with missense mutations) were also notable in the cell line models (Fig. 5e).

### CD19-CAR T cells revealed potent ex vivo activity against *RUNX1*^mut^ BP-CML patient cells with an additive effect to TKI inhibition

**Fig. 6:**
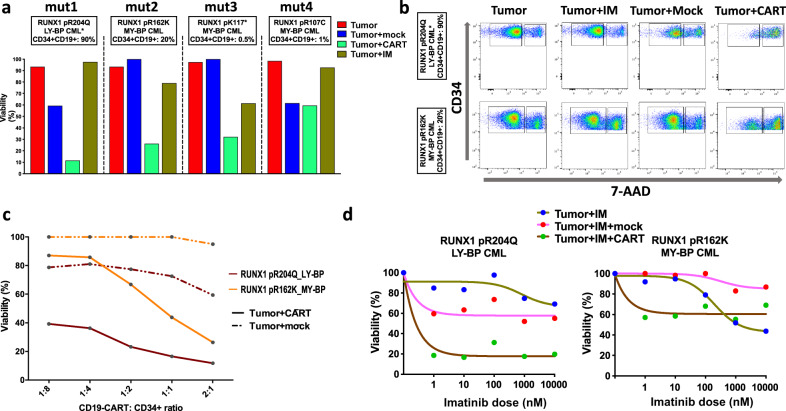
Ex vivo CD19-CAR T-cell activity against *RUNX1*^mut^ BP-CML patient blasts. **a** Comparison of ex vivo activity of CD19-CAR T cells, mock-CAR T cells, and imatinib (100 uM) and combination of imatinib and CAR T cells in *RUNX1*^mut^ BP-CML patients (*n* = 4) after 24-h incubation (effector–target, E–T ratio 2:1). The upper tags show RUNX1 mutations, BP phenotype, and percentage of CD34+CD19+ out of blast cells. Bar height represent viability percentage. CD19-CAR T cells showed the highest activity in lymphoid-BP patient (mut1) with T315I resistance mutation compared with mock cells and imatinib. They also had comparable activity as imatinib in myeloid-BP patients with more cytotoxic activity in patient mut2 with aberrant CD19 expression (20% of the cells). **b** Flow cytometry plot showing the activities of imatinib (IM), mock and CD19-CAR T cells (E–T ratio 2:1) on CD34+ blasts in two *RUNX1*^mut^ BP-CML patients (lymphoid BP (mut1) in the upper panel and myeloid BP (mut2) with aberrant CD19 in the lower panel) after 24-h incubation. Each plot is constructed by plotting 7-AAD expression on the *X*-axis and CD34 on *Y*-axis, with the right gate showing dead cells (7-AAD positive) and the left gate showing viable cells (7-AAD negative). CD19-CAR T cells were capable of inducing potent killing of blasts in both patients. **c** Comparison of mock and CD19-CAR T cells activities using different E–T ratios in two BP-CML patients (mut1 and mut2). CD19-CAR T cells at concentrations as low as 1:8 blasts were able to induce blast killing. Non-specific killing activity by mock cells was observed in ly-BP patient (mut1) but not in my-BP patient (mut2). **d** Dose–response curves of imatinib in serial concentration (range 1–10000 μM, 5 concentrations) alone and in combination with mock and CD19-CAR T cells (at 1:2 E–T ratio) in two BP patients after 24-h incubation. Ly-BP (mut1) with T315I mutation showed resistance to imatinib but potent activity to CD19-CAR T cells + imatinib combination. In my-BP patient (mut2), CD19-CAR T cells showed additive killing effect to imatinib at low imatinib concentrations.

Given the *RUNX1*^mut^-associated distinct phenotype, namely the aberrant expression of CD19 lymphoid marker in myeloid blast cells, we investigated the potential use of CD19-CAR T-cell immunotherapy in *RUNX1*^mut^ BP-CML patients. We tested the ex vivo cytotoxic activity of CD19-CAR T cells against *RUNX1*^mut^ BP-CML blasts (i.e., CD34-positive cells) with and without imatinib using flow cytometry (Fig. 6a,b and Supplementary Fig. 9a). CD19-CAR T cells showed a potent activity against *RUNX1*^mut^ BP-CML blasts in patients expressing CD19 including one lymphoid-BP (mut1) and one myeloid-BP patient (mut2) with aberrant CD19 expression on 25% of blasts. In a 24-h coculture experiment, CD19-CAR T cells were able to induce killing of blasts at effector–target (E–T) (CAR T cells: CD34+ cell) ratio as low as 1:8 at a variable extent (13–50%). The demonstrated cytotoxic activity of CD19-CAR T cells was specific in contrast with mock-CAR T cells (Fig. 6c). At an E–T ratio of 2:1, CD19-CAR T cells-induced killing was superior to killing by imatinib (100 nM), not only in mut1 patient who carried *ABL1-T315I* resistance mutation, but also in mut2 with no TKI-resistance mutation. Combining CD19-CAR T cells with imatinib showed an enhanced inhibitory effect compared with imatinib alone, or imatinib with mock cells (10,000-fold concentration range 1–10,000 nM) (Fig. 6d). Coculture of CD19-CAR T cells with blasts induced strong CD8+ CAR T cells activation, as demonstrated by 1.5–4-folds increase in CD69 expression. On the other hand, a modest activation of CD4+ CAR T cells was observed, as well as a minimal non-specific activation of mock-CAR T cells (Supplementary Fig. 9b).

In CD19-neg *RUNX1*-mutated patients (*n* = 2), CD19-CAR T cells-induced cytotoxicity was modest, highlighting the specificity of CAR T cells activity (Fig. 6a and Supplementary Fig. 10a). CD19-CAR T cells could still induce variable killing effect on BP-CML blasts, compared with mock-CAR T cells. Notably, in one myeloid-BP patient (mut4), CD19-CAR T-cells-induced cytotoxicity was superior to imatinib-induced inhibition (Supplementary Fig. 10a–c). In *RUNX1*^wt^ myeloid-BP patients, no enhanced activity with imatinib was noted, but cells from patient with CD19+ lymphoid-BP were killed effectively by CD19+ CAR T cells (Supplementary Fig. 10d).

## Discussion

Several studies have linked CML progression to the accumulation of somatic mutations and copy number changes [23–25]. However, whether these additional genetic aberrations define specific disease subtypes, which are still largely uncharacterized. In this work, we systematically studied the genomic, transcriptional, and drug sensitivity profiles of BP-CML primary patient samples with and without *RUNX1* mutations. Our study coupled *RUNX1* mutations in BP-CML with recombination events caused by off-target activity of AID/RAG complex. To our knowledge, this is the first such report in myeloid malignancies. Our results also highlighted the unique transcriptional and phenotypic signatures of *RUNX1*^mut^ BP-CML patients with aberrant expression of lymphoid markers including CD19. Finally, we demonstrated a potential role for the CAR T-cells immunotherapy in addition to targeted therapy in *RUNX1*^mut^ BP-CML patients.

The incidence of *RUNX1* mutations in BP-CML patients ranges between 12.9 and 33.3%, varying with the cohort size, disease phenotype (myeloid or lymphoid), and the sequencing method used [22, 24, 27]. Recently in a large BP-CML cohort [33], *ABL1* and *RUNX1* mutations were the most common mutations. In our own discovery cohort, we identified four mutations of *RUNX1*, that were located within the Runt domain, in line with reports of BP-CML and AML [11, 26, 27]. The identified variants have been reported in AML, displaying variable effects on *RUNX1* protein functions, including CBFB dimerization and DNA binding, in addition to leukemia transformation [34]. In a myeloid-BP patient, the *RUNX1* mutation was the sole leukemia-associated mutation identified both in CP (SNV) and progression (SNV and LOH) samples. Giustacchini et al. [35]. similarly reported a *RUNX1* mutation in both CP stem cells (SCs) and BP-SCs of a lymphoid-BP patient. *RUNX1*^mut^ CP-SCs demonstrate transcriptional similarities with BP-SCs, rather than with *RUNX1*^wt^ CP-SCs.

*RUNX1* aberrations contribute to mutagenesis and leukemic predisposition [36], and associate with downregulation of DNA repair genes in AML [37]. Likewise, we demonstrated downregulation of DNA repair genes, including *CETN2* and *MLH1*, in *RUNX1*^mut^ BP-CML. We identified *RAG*-mediated recombination to be associated with *RUNX1*^mut^. *RUNX1* is important for *RAG* function in early T-cell development [38]. Aberrant *AID/RAG* activity is implicated in lymphoid malignancies, namely in *ETV6-RUNX1* ALL [8]. Our data revealed transcriptional upregulation of several components of *AID/RAG*, which can increase genetic vulnerability [39]. Leukemia cells from *RUNX1*^mut^ patients exhibited significant association with *AID/RAG*-related SHM signature and enrichment of RAG-RSS compared with cells from *RUNX1*^wt^ patients. We demonstrated presence of RAG off-target activity in an *IKZF1* deletion in *RUNX1*^mut^ BP-CML, like previously reported in Philadelphia-positive ALL (Ph-ALL) [40]. *AID* expression was suggested to contribute to lymphoid progression in CML [41]. Recently, Thomson et al. reported that RAG off-target activity plays a central role in the progression of lymphoid-BP patients [42]. Interestingly, they reported a *RUNX1*^mut^ myeloid-BP patient with exceptionally high RAG expression and aberrant lymphoid markers phenotype supporting the role of *RUNX1*^mut^-induced RAG activity.

*RUNX1*^mut^ BP-CML shares several genomic features with *RUNX1*^mut^ AML, underscoring similarity of *RUNX1*^mut^ across leukemias. *RUNX1*^mut^ BP-CML exhibited other mutations in *BCORL1* and *PHF6* genes, as well as *IKZF1* deletions, comparable to the mutational landscape of *RUNX1*^mut^ AML [20, 21, 43]. In addition, upregulation of early HSC signature, lymphoid markers, and various AML prognostic markers in *RUNX1*^mut^ BP-CML was another similarity with *RUNX1*^mut^ AML [44, 45]. Downregulation of the coagulation pathway and megakaryocytic markers is consistent with the role of *RUNX1* mutations in FPD/AML [46]. Furthermore, *RUNX1*^mut^ BP-CML showed aberrant expression of lymphoid antigens (CD19, CD7) in myeloid-BP patients and overexpression of lymphoid TFs and markers similar to *RUNX1*^mut^ AML [47]. Aberrant expression of CD19 has been described in t*(8;21)-*AML to relate with *PAX5* overexpression [17]. We demonstrated overexpression of *PAX5* in *RUNX1*^mut^ BP-CML patients, in concord with data from *RUNX1*^mut^ AML [48]. *RUNX1*^mut^ BP-CML patients showed upregulation of many pDC markers. *RUNX1* is a key TF in pDC development through regulation of *IRF8* [49]. A recently described AML entity, “AML with pDC differentiation” [50], demonstrated frequent *RUNX1* mutations and expression of lymphoid antigens, comparable to *RUNX1*^mut^ BP-CML. Noteworthy, a study including 47 *RUNX1*^mut^-AML patients showed that *RUNX1*^mut^ blasts shared a common gene expression signature in contrast with *RUNX1*^wt^ blasts, and transcriptional differences between missense and nonsense *RUNX1* mutations were demonstrated in some *RUNX1* target genes [45]. Further studies and analysis of recently published data [33] will add to our understanding of mutation-specific-induced transcriptional changes in BP-CML.

Development of new therapeutic options is essential for management of BP-CML [51]. We identified potentially useful targeted drugs for *RUNX1*^mut^ BP-CML patients, including mTOR inhibitors, glucocorticoids, VEGFR inhibitors, and BCL2 inhibitors. VEGFR and mTOR inhibitors are active in CBF-AML [52, 53]. Likewise, glucocorticoids [45] and BCL2 inhibitors [54] showed inhibitory effects in *RUNX1*^mut^AML, reflecting shared *RUNX1*^mut^ signature. In *RUNX1*^mut^AML, glucocorticoid sensitivity is associated with *RUNX1* mutations and wild-type RUNX1 activity [45], which potentially explains variances in glucocorticoid activity in our samples also. BET inhibitors that demonstrate selective activity in *RUNX1*^mut^ cell lines were recently suggested as a targeted therapy for *RUNX1*^mut^ AML [55]. Combination of imatinib with the selected drugs displayed synergistic inhibition of *RUNX1*^mut^ blasts, representing promising treatment strategies for *RUNX1*^mut^ BP-CML. A strong evidence on the selective sensitivity of *RUNX1*^mut^ blasts was also demonstrated in our previous study where a lymphoid-BP patient with a dominant *RUNX1*^mut^ clone (VAF:48%) received a DSRT-based VEGFR inhibitor axitinib, which yielded clearance of the *RUNX1*^mut^ clone at relapse [25].

Our study also highlighted immunotherapy as an effective approach for BP-CML management, especially in *RUNX1*^mut^ BP-CML patients with CD19 expression. *RUNX1* mutations are associated with upregulation of several molecular targets for immunotherapy, including CD19 [56] and CD133 [57]. CD19-CAR T-cell therapy has been implemented in management of B-cell lymphomas, ALL, and Ph-ALL patients [58]. Combination of CAR T cells with other immunotherapeutic approaches or targeted therapies can further improve response rates to CAR T cells treatment [59, 60]. We demonstrated a potent ex vivo cytotoxic targeting of CD19-CAR T cells against *RUNX1*^mut^ BP-CML blasts in both myeloid-BP and lymphoid-BP patients. In combination with imatinib, CD19-CAR T cells showed enhanced killing of *RUNX1*^mut^ BP-CML blasts. CD19-CAR T cells successfully targeted imatinib-resistant blasts, highlighting CD19-CAR T cells as a potentially effective strategy in BP-CML specially CD19-positive *RUNX1*^mut^ BP-CML patients. The therapeutic approach combining TKIs and CD19-CAR T cells may also reduce the possibility of CD19-neg relapses, previously encountered both with CD19 targeting antibodies and CD19-CAR T cells in ALL [61, 62]. Interestingly, recent case report described that CD19-CAR T cells are able to induce remission in t(8;21) AML patient [63] suggesting that in addition to BP-CML, this therapy modality could be effective in other *RUNX1*^mut^ leukemia.

In conclusion, this study provides insights into the role of *RUNX1* mutations in CML progression by induced transcriptional reprogramming and aberrant mutagenic AID/RAG activity. Employing comprehensive phenotypic, genetic, transcriptional, and drug sensitivity profiling data highlighted multiple deregulated signaling pathways that represent novel options for targeted therapy, and together with CD19-CAR T-cell immunotherapeutic approach, may provide a means to improve management of poor prognosis BP-CML patients.

## Supplementary information

Supplemental material

Supplemental dataset 1

Supplemental dataset 2

Supplemental dataset 3

Supplemental dataset 4

Supplemental dataset 5

Supplemental dataset 6

Supplemental dataset 7

Supplemental dataset 8

Supplemental dataset 9

Supplemental dataset 10

## Data Availability

WES and RNA-sequencing data and deidentified individual participant data are available from the corresponding author upon request.

## References

[1] Imperato MR, Cauchy P, Obier N, Bonifer C (2015). The RUNX1-PU.1 axis in the control of hematopoiesis. Int J Hematol.

[2] Sood R, Kamikubo Y, Liu P (2017). Role of RUNX1 in hematological malignancies. Blood.

[3] Churpek JE, Pyrtel K, Kanchi K-L, Shao J, Koboldt D, Miller CA (2015). Genomic analysis of germ line and somatic variants in familial myelodysplasia/acute myeloid leukemia. Blood.

[4] De Braekeleer E, Douet-Guilbert N, Morel F, Le Bris M-J, Férec C, De Braekeleer M (2011). RUNX1 translocations and fusion genes in malignant hemopathies. Future Oncol.

[5] Peterson LF, Zhang D-E (2004). The 8;21 translocation in leukemogenesis. Oncogene.

[6] Becker M, Liu K, Tirado CA (2017). The t(12;21)(p13;q22) in pediatric B-acute lymphoblastic leukemia: an update. J Assoc Genet Technol.

[7] Li S, Yin CC, Medeiros LJ, Bueso-Ramos C, Lu G, Lin P (2012). Myelodysplastic syndrome/acute myeloid leukemia with t(3;21)(q26.2;q22) is commonly a therapy-related disease associated with poor outcome. Am J Clin Pathol.

[8] Papaemmanuil E, Rapado I, Li Y, Potter NE, Wedge DC, Tubio J (2014). RAG-mediated recombination is the predominant driver of oncogenic rearrangement in ETV6-RUNX1 acute lymphoblastic leukemia. Nat Genet.

[9] Muramatsu M, Kinoshita K, Fagarasan S, Yamada S, Shinkai Y, Honjo T (2000). Class switch recombination and hypermutation require activation-induced cytidine deaminase (AID), a potential RNA editing enzyme. Cell.

[10] Oettinger MA, Schatz DG, Gorka C, Baltimore D (1990). RAG-1 and RAG-2, adjacent genes that synergistically activate V(D)J recombination. Science.

[11] Tang J-L, Hou H-A, Chen C-Y, Liu C-Y, Chou W-C, Tseng M-H (2009). AML1/RUNX1 mutations in 470 adult patients with de novo acute myeloid leukemia: prognostic implication and interaction with other gene alterations. Blood.

[12] Grossmann V, Kern W, Harbich S, Alpermann T, Jeromin S, Schnittger S (2011). Prognostic relevance of RUNX1 mutations in T-cell acute lymphoblastic leukemia. Haematologica.

[13] Chen C-Y, Lin L-I, Tang J-L, Ko B-S, Tsay W, Chou W-C (2007). RUNX1 gene mutation in primary myelodysplastic syndrome–the mutation can be detected early at diagnosis or acquired during disease progression and is associated with poor outcome. Br J Haematol.

[14] Patnaik MM, Tefferi A (2016). Cytogenetic and molecular abnormalities in chronic myelomonocytic leukemia. Blood Cancer J.

[15] Yamato G, Shiba N, Yoshida K, Hara Y, Shiraishi Y, Ohki K (2018). RUNX1 mutations in pediatric acute myeloid leukemia are associated with distinct genetic features and an inferior prognosis. Blood.

[16] Silva FPG, Swagemakers SMA, Erpelinck-Verschueren C, Wouters BJ, Delwel R, Vrieling H (2009). Gene expression profiling of minimally differentiated acute myeloid leukemia: M0 is a distinct entity subdivided by RUNX1 mutation status. Blood.

[17] Walter K, Cockerill PN, Barlow R, Clarke D, Hoogenkamp M, Follows GA (2010). Aberrant expression of CD19 in AML with t(8;21) involves a poised chromatin structure and PAX5. Oncogene.

[18] Plesa A, Labussière-Wallet H, Hayette S, Salles G, Thomas X, Sujobert P (2019). Efficiency of blinatumomab in a t(8;21) acute myeloid leukemia expressing CD19. Haematologica.

[19] Schnittger S, Dicker F, Kern W, Wendland N, Sundermann J, Alpermann T (2011). RUNX1 mutations are frequent in de novo AML with noncomplex karyotype and confer an unfavorable prognosis. Blood.

[20] Haferlach T, Stengel A, Eckstein S, Perglerová K, Alpermann T, Kern W (2016). The new provisional WHO entity “RUNX1 mutated AML” shows specific genetics but no prognostic influence of dysplasia. Leukemia.

[21] Gaidzik VI, Teleanu V, Papaemmanuil E, Weber D, Paschka P, Hahn J (2016). RUNX1 mutations in acute myeloid leukemia are associated with distinct clinico-pathologic and genetic features. Leukemia.

[22] Grossmann V, Kohlmann A, Zenger M, Schindela S, Eder C, Weissmann S (2011). A deep-sequencing study of chronic myeloid leukemia patients in blast crisis (BC-CML) detects mutations in 76.9% of cases. Leukemia.

[23] Branford S, Kim DDH, Apperley JF, Eide CA, Mustjoki S, Ong ST (2019). Laying the foundation for genomically-based risk assessment in chronic myeloid leukemia. Leukemia.

[24] Branford S, Wang P, Yeung DT, Thomson D, Purins A, Wadham C (2018). Integrative genomic analysis reveals cancer-associated mutations at diagnosis of CML in patients with high-risk disease. Blood.

[25] Adnan Awad S, Kankainen M, Ojala T, Koskenvesa P, Eldfors S, Ghimire B (2020). Mutation accumulation in cancer genes relates to nonoptimal outcome in chronic myeloid leukemia. Blood Adv.

[26] Yamamoto K, Tsuzuki S, Minami Y, Yamamoto Y, Abe A, Ohshima K (2013). Functionally deregulated AML1/RUNX1 cooperates with BCR-ABL to induce a blastic phase-like phenotype of chronic myelogenous leukemia in mice. PLOS One.

[27] Zhao L-J, Wang Y-Y, Li G, Ma L-Y, Xiong S-M, Weng X-Q (2012). Functional features of RUNX1 mutants in acute transformation of chronic myeloid leukemia and their contribution to inducing murine full-blown leukemia. Blood.

[28] Arber DA, Orazi A, Hasserjian R, Thiele J, Borowitz MJ, Beau MML (2016). The 2016 revision to the World Health Organization classification of myeloid neoplasms and acute leukemia. Blood.

[29] Pemovska T, Kontro M, Yadav B, Edgren H, Eldfors S, Szwajda A (2013). Individualized systems medicine strategy to tailor treatments for patients with chemorefractory acute myeloid leukemia. Cancer Discov.

[30] Kaartinen T, Luostarinen A, Maliniemi P, Keto J, Arvas M, Belt H (2017). Low interleukin-2 concentration favors generation of early memory T cells over effector phenotypes during chimeric antigen receptor T-cell expansion. Cytotherapy.

[31] Dufva O, Koski J, Maliniemi P, Ianevski A, Klievink J, Leitner J (2020). Integrated drug profiling and CRISPR screening identify essential pathways for CAR T cell cytotoxicity. Blood.

[32] Alexandrov LB, Nik-Zainal S, Wedge DC, Aparicio SAJR, Behjati S, Biankin AV (2013). Signatures of mutational processes in human cancer. Nature.

[33] Ko TK, Javed A, Lee KL, Pathiraja TN, Liu X, Malik S (2020). An integrative model of pathway convergence in genetically heterogeneous blast crisis chronic myeloid leukemia. Blood.

[34] Tsai S-C, Shih L-Y, Liang S-T, Huang Y-J, Kuo M-C, Huang C-F (2015). Biological activities of RUNX1 mutants predict secondary acute leukemia transformation from chronic myelomonocytic leukemia and myelodysplastic syndromes. Clin Cancer Res.

[35] Giustacchini A, Thongjuea S, Barkas N, Woll PS, Povinelli BJ, Booth CAG (2017). Single-cell transcriptomics uncovers distinct molecular signatures of stem cells in chronic myeloid leukemia. Nat Med.

[36] Bera R, Chiu M-C, Huang Y-J, Lin T-H, Kuo M-C, Shih L-Y (2019). RUNX1 mutations promote leukemogenesis of myeloid malignancies in ASXL1-mutated leukemia. J Hematol Oncol.

[37] Forster VJ, Nahari MH, Martinez-Soria N, Bradburn AK, Ptasinska A, Assi SA (2016). The leukemia-associated RUNX1/ETO oncoprotein confers a mutator phenotype. Leukemia.

[38] Cieslak A, Le Noir S, Trinquand A, Lhermitte L, Franchini D-M, Villarese P (2014). RUNX1-dependent RAG1 deposition instigates human TCR-δ locus rearrangement. J Exp Med.

[39] Swaminathan S, Klemm L, Park E, Papaemmanuil E, Ford A, Kweon S-M (2015). Mechanisms of clonal evolution in childhood acute lymphoblastic leukemia. Nat Immunol.

[40] Dong Y, Liu F, Wu C, Li S, Zhao X, Zhang P (2016). Illegitimate RAG-mediated recombination events are involved in IKZF1 Δ3-6 deletion in BCR-ABL1 lymphoblastic leukaemia. Clin Exp Immunol.

[41] Klemm L, Duy C, Iacobucci I, Kuchen S, von Levetzow G, Feldhahn N (2009). The B cell mutator AID promotes B lymphoid blast crisis and drug resistance in chronic myeloid leukemia. Cancer Cell.

[42] Thomson DW, Shahrin NH, Wang PPS, Wadham C, Shanmuganathan N, Scott HS (2020). Aberrant RAG-mediated recombination contributes to multiple structural rearrangements in lymphoid blast crisis of chronic myeloid leukemia. Leukemia.

[43] Stengel A, Kern W, Meggendorfer M, Nadarajah N, Perglerovà K, Haferlach T (2018). Number of *RUNX1* mutations, wild-type allele loss and additional mutations impact on prognosis in adult *RUNX1*-mutated AML. Leukemia.

[44] Assi SA, Imperato MR, Coleman DJL, Pickin A, Potluri S, Ptasinska A (2019). Subtype-specific regulatory network rewiring in acute myeloid leukemia. Nat Genet.

[45] Simon L, Lavallée V-P, Bordeleau M-E, Krosl J, Baccelli I, Boucher G (2017). Chemogenomic landscape of RUNX1-mutated AML reveals importance of RUNX1 allele dosage in genetics and glucocorticoid sensitivity. Clin Cancer Res.

[46] Iizuka H, Kagoya Y, Kataoka K, Yoshimi A, Miyauchi M, Taoka K (2015). Targeted gene correction of RUNX1 in induced pluripotent stem cells derived from familial platelet disorder with propensity to myeloid malignancy restores normal megakaryopoiesis. Exp Hematol.

[47] Greif PA, Konstandin NP, Metzeler KH, Herold T, Pasalic Z, Ksienzyk B (2012). RUNX1 mutations in cytogenetically normal acute myeloid leukemia are associated with a poor prognosis and up-regulation of lymphoid genes. Haematologica.

[48] Menter T, Lundberg P, Wenzel F, Dirks J, Fernandez P, Friess D (2019). RUNX1 mutations can lead to aberrant expression of CD79a and PAX5 in acute myelogenous leukemias: a potential diagnostic pitfall. Pathobiology.

[49] Satpathy AT, Briseño CG, Cai X, Michael DG, Chou C, Hsiung S (2014). Runx1 and Cbfβ regulate the development of Flt3+ dendritic cell progenitors and restrict myeloproliferative disorder. Blood.

[50] Xiao W, Goldberg AD, Famulare C, Baik J, Gao Q, Tallman MS, et al. Acute myeloid leukemia with plasmacytoid dendritic cell differentiation: predominantly secondary AML, enriched for RUNX1 mutations, frequent cross-lineage antigen expression and poor prognosis. Blood 2018;132 Suppl 1:2789–2789.

[51] Hehlmann R, Saußele S, Voskanyan A, Silver RT (2016). Management of CML-blast crisis. Best Pract Res Clin Haematol.

[52] Imai N, Shikami M, Miwa H, Suganuma K, Hiramatsu A, Watarai M (2006). t(8;21) acute myeloid leukaemia cells are dependent on vascular endothelial growth factor (VEGF)/VEGF receptor type2 pathway and phosphorylation of Akt. Br J Haematol.

[53] Fuka G, Kantner H-P, Grausenburger R, Inthal A, Bauer E, Krapf G (2012). Silencing of ETV6/RUNX1 abrogates PI3K/AKT/mTOR signaling and impairs reconstitution of leukemia in xenografts. Leukemia.

[54] Tyner JW, Tognon CE, Bottomly D, Wilmot B, Kurtz SE, Savage SL (2018). Functional genomic landscape of acute myeloid leukaemia. Nature.

[55] Mill CP, Fiskus W, DiNardo CD, Qian Y, Raina K, Rajapakshe K (2019). RUNX1-targeted therapy for AML expressing somatic or germline mutation in RUNX1. Blood.

[56] Filley AC, Henriquez M, Dey M (2018). CART immunotherapy: development, success, and translation to malignant gliomas and other solid tumors. Front Oncol.

[57] Bueno C, Velasco-Hernandez T, Gutiérrez-Agüera F, Zanetti SR, Baroni ML, Sánchez-Martínez D (2019). CD133-directed CAR T-cells for MLL leukemia: on-target, off-tumor myeloablative toxicity. Leukemia.

[58] Orlowski RJ, Porter DL, Frey NV (2017). The promise of chimeric antigen receptor T cells (CARTs) in leukaemia. Br J Haematol.

[59] Bernabei L, Garfall AL, Melenhorst JJ, Lacey SF, Stadtmauer EA, Vogl DT, et al. PD-1 inhibitor combinations as salvage therapy for relapsed/refractory multiple myeloma (MM) patients progressing after BCMA-directed CAR T cells. Blood 2018;132 Suppl 1:1973–1973.

[60] Gill SI, Vides V, Frey NV, Metzger S, O’Brien M, Hexner E, et al. Prospective clinical trial of anti-CD19 CAR T cells in combination with ibrutinib for the treatment of chronic lymphocytic leukemia shows a high response rate. Blood 2018;132 Suppl 1:298–298.

[61] Nagel I, Bartels M, Duell J, Oberg H-H, Ussat S, Bruckmueller H (2017). Hematopoietic stem cell involvement in BCR-ABL1-positive ALL as a potential mechanism of resistance to blinatumomab therapy. Blood.

[62] Pan J, Tan Y, Deng B, Tong C, Hua L, Ling Z, et al. Frequent occurrence of CD19-negative relapse after CD19 CAR T and consolidation therapy in 14 TP53-mutated r/r B-ALL children. Leukemia 2020. 10.1038/s41375-020-0831-z. 10.1038/s41375-020-0831-z 32346068

[63] Danylesko I, Jacoby E, Yerushalmi R, Shem-Tov N, Besser MJ, Vernitsky H (2020). Remission of acute myeloid leukemia with t(8;21) following CD19 CAR T-cells. Leukemia.

