# Supplementary material for: RUNX1 mutations in blast-phase chronic myeloid leukemia associate with distinct phenotypes, transcriptional profiles, and drug responses
Source: Leukemia. 2020 Aug 11;35(4):1087–99. doi: 10.1038/s41375-020-01011-5 (PMC8024199; doi:10.1038/s41375-020-01011-5)
Supplement: Supplementary file 1 — Supplemental material [file 41375_2020_1011_MOESM1_ESM.docx]

***RUNX1* Mutations in Blast Phase Chronic Myeloid Leukemia Associate with Distinct Phenotypes, Transcriptional Profiles, and Drug Responses**

**Running title:** *RUNX1* mutations in Blast Phase CML

Shady Adnan Awad^1,2,3^, Olli Dufva^1,2,4^ Aleksandr Ianevski^5,6^, Bishwa Ghimire^5^, Jan Koski^7^, Pilvi Maliniemi^7^, Daniel Thomson^8,9^, Andreas Schreiber^9,10,11^, Caroline A. Heckman^5^, Perttu Koskenvesa^1^, Matti Korhonen^7^, Kimmo Porkka^1,2,4^, Susan Branford^8,9^, Tero Aittokallio^4,5,6,12^, Matti Kankainen^1,2,4*^, Satu Mustjoki^1,2,4*^

^1^Hematology Research Unit Helsinki, University of Helsinki and Helsinki University Hospital Comprehensive Cancer Center, Helsinki, Finland ^2^ Translational Immunology Research Program and Department of Clinical Chemistry and Hematology, University of Helsinki, Helsinki, Finland ^3^Clinical pathology department, National Cancer Institute, Cairo University, Cairo, Egypt ^4^iCAN Digital Precision Cancer Medicine Flagship ^5^Institute for Molecular Medicine Finland (FIMM), Helsinki Institute of Life Science (HiLIFE), University of Helsinki, Helsinki, Finland ^6^Helsinki Institute for Information Technology (HIIT), Department of Computer Science, Aalto University, Espoo, Finland ^7^Finnish Red Cross Blood Service, Helsinki, Finland ^8^Department of Genetics and Molecular Pathology, Centre for Cancer Biology, SA Pathology, Adelaide, Australia ^9^School of Pharmacy and Medical Science, Division of Health Sciences, University of South Australia, Adelaide, Australia ^10^Australian Cancer Research Foundation Genomics Facility, Centre for Cancer Biology, SA Pathology, Adelaide, Australia ^11^School of Biological Sciences, University of Adelaide, Adelaide, Australia ^12^Department of Mathematics and Statistics, University of Turku, Turku, Finland * Authors contributed equally to this work

**Corresponding author**: Prof. Satu Mustjoki, Hematology Research Unit Helsinki, University of Helsinki and Helsinki University Hospital Comprehensive Cancer Center, Haartmaninkatu 8, P.O. Box 700, FIN-00290 Helsinki, Finland. Tel: +358 9 471 71898, Fax: +358 9 471 71897, e-mail: satu.mustjoki@helsinki.fi

**Supplementary Figures**

**Supplementary figure 1. Mutational profile of *RUNX1*^mut^ BP-CML patients.** a) Landscape of mutations in *RUNX1*-mutated BP-CML from 3 cohorts (this study, Branford et al 2018(1) and Grossmann et al 2012(2)). Explanatory tracks from top to bottom show phenotype of the accelerated phase (AP)/blast phase (BP) cases and sequencing method. The filling color indicates the type of the variant. Chromosomal abnormalities are shown in the lower part of the plot. The bottom explanatory track indicates the study cohort. b) Immunophenotyping analysis of BM cells from *RUNX1* pR162K myeloid-BP patient showed characteristic phenotype: aberrant CD19 expression (16% of CD34+ cells) and relatively high pDC fraction (CD123+ HLA-DR+). Flow data from all patients are shown in Supplementary table 1. c) Fish plot showing the clonal evolution of a *RUNX1* pR162K myeloid-BP patient during transformation from diagnosis/CP (blast level 4%) to BP (blast level 65%). Clonal analysis highlighted expansion of RUNX1 mutated clone with LOH in 21q and loss of the normal RUNX1 (*RUNX1* pR162K VAF:99%) and acquisition of additional mutations ex: SCUBE2.

**Supplementary figure 2. Combined data of BP-CML patients.** a) Comparison of the mutational load as calculated by the number of SNVs per mbp per sample between *RUNX1*^mut^ (n=7) and *RUNX1*^wt^ (n=13) BP-CML patients, (median= 0.415 mutations/mbp and 0.343 mutations/mbp respectively, *p=ns*) b) Comparison of the SVs count as calculated by the number of SVs per sample between *RUNX1*^mut^ (n=7) and *RUNX1*^wt^ (n=13) BP-CML patients, (median 3 SVs/sample and 2 SVs/sample respectively, *p=ns*) c) Correlation matrix showing Spearman correlation between normalized weights of signatures of *RUNX1*^mut^ and *RUNX1*^wt^ BP-CML patients from our cohort (FIN), Australian cohort (AUS) and combined data. Blue dot color indicates positive correlations and red negative correlations as expressed in r values. The dot size represents -log10 *p*-values. **d**) Number of SNVs identified in each sample by the consequence of the mutation, **e**) number of small insertion and deletions identified in each sample by the consequence of the frameshift insertion and deletion, and **f**) number of SVs identified in each sample by the type of the mutation.

**Supplementary figure 3. Motif search in BP-CML patients.** Results of motif search performed using standard MEME tool (see online methods) at different padding length (output motif limits) for breakpoints in *RUNX1*^mut^ and *RUNX1*^wt^ BP-CML patients.

**Supplementary figure 4.: Expression profiles of *RUNX1*^mut^ BP-CML patients. a)** Heatmap of the expression level of top 200 variably expressed genes among BP samples (n=9). The explanatory track from above are indicating RUNX1 mutation status, blast phenotype and predicted effect of mutation. The right panel shows genes which were found as statistically differentially expressed between *RUNX1*^mut^ and *RUNX1^wild^* samples (Q-value ≤0.05). b) Bar plots showing the expression levels of selected genes that were significantly differentially expressed between *RUNX1*^mut^ and *RUNX1*^wt^ BP-CML patients, including stem cell genes (*CD133, CD34, CD25*), lymphoid TFs and markers (*PAX5, VPERB1, CD19*), pDC TFs and markers (*IRF8, TCF4, CIITA*) and downregulated myeloid and megakaryocytic TFs (*ITG3B, PF4, PPBP*). * *p* <0.05, ** *p*<0.01, *** *p*<0.001, two-tailed student’s t-test, Q-value ≤0.05 for all genes except *CD34* (Q-value ≤0.063).

**Supplementary figure 5.: Expression profiles of *RUNX1*^mut^ lymphoid BP-CML patients from Branford S. et al paper. a)**  PCA plot showing clustering of lymphoid-BP samples (n=16, *RUNX1*^mut^ =7, *RUNX1*^wt^ =9) according to *RUNX1* mutation status. **b)** Heatmap of the expression level of top 100 differentially expressed genes among lymphoid-BP samples (n=16). The explanatory track from above are indicating RUNX1 mutation status. Data derived from Branford et al, Blood 2018(1). **c**) GSEA of CBF-AML and AML with t(8;21) pathways upregulated in *RUNX1*^mut^ compared to *RUNX1*^wt^ patients.

**Supplementary figure 6.: Drug response profiles in association with *RUNX1* mutations.** a) The top 20 most sensitive targeted compounds across *RUNX1^mut^* BP-CML patient samples ranked by the median DSS scores*. C*onventional chemotherapeutic drugs (Supplementary Table 8) and broadly active compounds (CUDC-907, KX2-391, UCN-01, ONX-0914) are excluded. b) Correlation of DSS scores between *RUNX1*^mut^ (n=3) and *RUNX1^wild^* (n=3) samples from myeloid BP-patients only (2 lymphoid-BP samples are excluded). Comparison highlighted specific activity of glucocorticoids, mTOR, VEGFR, MEK inhibitors in addition to navitoclax in *RUNX1*^mut^ samples. c) Comparison of sensitivity to methylprednisolone (glucocorticoids), temsirolimus (mTOR inhibitor), axitinib (VEGFR inhibitor) and AZD8055 (mTOR inhibitor) between *RUNX1^mut^* and *RUNX1*^wt^ myeloid BP patient samples. d) Comparison of gene expression levels (CPM log_2_ values) of *NC3R1* (target of glucocorticoids, *Q<0.05*) and *FLT4* (target of VEGFR-inhibitors, *Q=0.144*) between *RUNX1^mut^* and *RUNX1*^wt^ samples (* *p* <0.05, ** *p*<0.01, *** *p*<0.001, two-tailed student’s t-test). e) Correlation of DSS scores between patients with nonsense (n=1) and missense (n=2) *RUNX1* mutations. Glucocorticoids and mTOR inhibitors show differentially higher activity in patient with nonsense mutation while navitoclax is showing higher activity in missense *RUNX1* mutated patients’ samples.

**Supplementary figure 7.: Drug combination in *RUNX1*^mut^ BP patients.** Dose response synergy heatmaps of drug combinations (n=5 combination of imatinib with either everolimus, cobimetinib, axitinib, dexamethasone and venetoclax) in *RUNX1^mut^* (n=3) and *RUNX1^wt^* (n=2) BP-CML patients. BP- phenotypes: my-BP: myeloid BP, lym-BP: lymphoid BP, amb-BP: ambiguous BP. Hsa synergy scores (see methods) are supplemented for each combination. Full lists of individual drug DSS, hsa synergy scores in Supplementary Table 8.

**Supplementary figure 8.: CRISPR-editing of Baf3-P210-BCR-ABL1 cell lines and induced phenotypic changes.** a) Sequencing of mouse *runx1*-exon 4 (upper panel) and human *RUNX1*-exon 2 (lower panel) target area showed efficient editing in *RUNX1^-/-^* and *RUNX1^-/mut^* lines compared to wild type control (*RUNX1^wt/wt^)* and parental cell line. b) Predicted effect of the CRISPR-editing on protein structure using TIDE tool(3) in mouse lines (upper panel) showed a homozygous frameshift (-1) deletion in *RUNX1^-/-^* line and heterozygous frameshift (-1) and in-frame (-3) deletions in *RUNX1^-/mut^* line. In human lines (lower panel), *RUNX1^-/-^* line showed multiple variable-sized indels with no trace of wild type clone. The bar height represents the predicted clone size fraction with the relevant indel and the position of the bar represents the type of induced indel (insertion (+)/deletion(-)) and the indel size (distance from the predicted breakpoint/PAM sequence). The predicted total CRISPR-editing efficiency is presented on the upper left corner of each figure. Comparison of drug responses of c) K562 *RUNX1^-/-^* line d) Baf3-BCR-ABL1 with induced expression of *RUNX1^R162K^* mutant and e) K562 with induced expression of *RUNX1^R162K^* mutant to selected agents NVP-LCL161, navitoclax, axitinib and AZD8055 in contrast with relevant parental lines. The mutant RUNX1 expression induced differential effect on cell lines which are similar to patients’ profiles (acquired resistance to LCL-161 in K562 and Baf3 cells, sensitivity to AZD8055 and axitinib in K562-*RUNX1^R162K^* and navitoclax in Baf3*-RUNX1^R162K^* cells). Both cell lines are inherently resistant to glucocorticoids and rapalogs.

**Supplementary figure 9. CD19-CAR T-cell activity in *RUNX1*^mut^ BP-CML patients.** a) Comparison of ex-vivo activity of CD19-CAR T-cells, mock-CAR T-cells, imatinib (100uM) and combination of imatinib and CAR T-cells in *RUNX1*^mut^ BP-CML patients (n=4) after 24h incubation (effector-target ratio 1:2). The upper tags show RUNX1 mutations, BP phenotype and percentage of CD34+CD19+ out of blast cells. The experiment was done in triplicates. Bar height represent mean viability percentage and error bars indicate standard deviation. CD19-CAR T-cells showed the highest activity in lymphoid BP patient (mut1) with T315I resistance mutation compared to mock cells and imatinib. CAR T cells also had comparable activity as imatinib in myeloid-BP patients with highest cytotoxic activity in patient mut2 with aberrant CD19 expression (20% of the cells). b) Comparison of CD69 expression (%) changes in mock and CD19-CAR T-cells (CD8 upper panel and CD4 lower panel) after a 24-hour incubation with *RUNX1*^mut^ BP-CML patients’ samples as a foldchange values relative to unstimulated cells (median CD8CD69+: 6%, and CD4CD69+: 57% in unstimulated cells.

**Supplementary figure 10. CD19-CAR T-cell activity in *RUNX1*^mut^ and *RUNX1*^wt^ BP-CML patients.** a) Flow cytometry plot showing the activities of imatinib (IM), mock and CD19-CAR T-cells on CD34+ blasts in 2 *RUNX1*^mut^ BP-CML patients (mut3 and mut4, myeloid BP with no CD19 expression) after a 24hour incubation at an E-T ratio 1:2. Each plot is constructed by plotting 7-AAD expression on the X axis and CD34 on Y axis, with the right gate showing dead cells (7-AAD positive) and the left gate showing viable cells (7-AADnegative). CD19-CAR T-cells are capable of inducing modest killing of blasts in both patients, even with the absence of CD19 expression on blast cells. b) Comparison of mock and CD19-CAR T-cells activities at different E:T ratios in 2 myeloid BP-CML patients (mut3 and mut4) showing specific activity of CD19-CAR T-cells in killing BP-CML blasts (indicated by percentage of viable cells after a 24hour incubation). No difference between mock and CD19-CAR T-cells killing were demonstrated in *RUNX1^R107C^* patient (mut4). In patient (mut3) with *RUNX1^K117*^,* killing activity was positively correlated with increasing CD19-CAR T-cells concentration while similar effect was not observed with mock cells, c) Drug responses curves of imatinib in serial concentrations (range between 1-10000 uM, 5 concentrations) alone and in combination with mock and CD19-CAR T-cells (at E-T ratio 1:2) in 2 BP patients after 24 hrs incubation. Patient (mut4) with *RUNX1^K117*^* showed specific and additive activity of imatinib+CD19-CAR T-cells combination specially at low IM concentrations. d) Comparison of ex-vivo activity of CD19-CAR T-cells, mock-CAR T-cells, imatinib (100uM) and combination of imatinib and CAR T-cells in *RUNX1*^wt^ BP-CML patients (n=4) after 24h incubation (E-T ratio 1:2). The upper tags show BP phenotype and percentage of CD34+CD19+ out of blast cells. The experiment was done in triplicates. Bar height represent mean viability percentage and error bars indicate standard deviation.

**Supplementary Tables:**

**Supplementary Table 1.** Clinicopathological features and flowcytometry data of BP-CML patients’ cohort.

**Supplementary Table 2.** Details of sgRNA primers, oligos and antibodies used in cell line experiments.

**Supplementary Table 3.** Details of antibodies panel used in CAR T-cell phenotyping and cytotoxicity experiments.

**Supplementary Table 4.** Mutations identified in all samples**.**

**Supplementary Table 5.** Results from structural rearrangement analysis.

**Supplementary Table 6.** Correlation of mutational signature profiles in CML patients’ subsets.

**Supplementary Table 7.** Differential expression analysis of RNA-sequencing data**.**

**Supplementary Table 8.** Drug sensitivity profiling data of BP-CML patients.

**Supplementary Table 9.** RNA-sequencing data of BCR-ABL1/Ba/f3 edited cell lines.

**Supplementary Table 10.** Drug sensitivity profiling data of CRISPR edited cell lines.

**Supplementary methods**

***Flowcytometry analysis***

Thawed bone marrow mononuclear cells (BMNCs) were stained for 15min with surface markers CD45-V500, CD34-FITC, CD3-Percp-Cy5.5, CD303/BDCA-APC, CD115-PE, CD123-BV421, CD19-APC-Cy7 and HLA-DR-PE-Cy7. For mouse cell lines, rat anti-mouse CD45-PE, CD19-PE and CD11b-BV510 were applied using recommended manufacturer protocols for surface antibodies staining. A minimum of 1x10^6^ cells were acquired with the FACSVerse and analyzed with FlowJo (Version 10.0.8r1, TreeStar) software. All of the antibodies were purchased from BD Biosciences (San Diego, CA, USA).

***RNA sequencing***

Total RNA was isolated from bone marrow cells of nine BP-CML cases using miRNeasy Mini Kit (Qiagen). RNA integrity was measured by Agilent Bioanalyzer RNApico chip (Agilent) and Qubit RNA kit (Life Technologies) to quantitate RNA in samples. We used 1.5 µg of total RNA for ScriptSeq v2™ Complete kit for human/mouse/rat (Illumina) to perform ribodepletion of rRNA and further for RNA-seq library preparation for all samples except for two BP-CML of which RNA-sequencing libraries were prepared using Illumina compatible Nextera™ Technology (Epicentre) and one BP sample that were prepared using Truseq standard total RNA preparation kit (illumina). RNA-sequencing libraries were purified using SPRI beads (Agencourt AMPure XP, Beckman Coulter). High Sensitivity chips by Agilent Bioanalyzer (Agilent) was used to evaluate the library quality. Paired-end sequencing of sequencing libraries was performed using Illumina HiSeq technology (HiSeq 2000, Illumina) (n=8) and Novaseq S2 XP NS2-200 (Illumina) (n=1 sample).

***Drug sensitivity and resistance testing (DSRT)***

The oncology compound library, employed to test patient samples, consisted of 125 FDA/EMA anti-cancer approved drugs and 127 investigational and preclinical compounds (Supplementary Table 8). For the cell lines, the full library of 528 compounds (156 approved drugs and 372 investigational compounds) were used. All drugs were purchased from commercial vendors and dissolved in either 100% dimethyl sulfoxide (DMSO) or water. DSRT was performed as earlier described(4). Briefly, each compound was tested in five different concentrations, covering a 10 000-fold concentration range, pre-printed on 384-microwell plates (Corning) with an acoustic liquid handling device (Echo 550, Labcyte Inc.). Five µl culture medium per well was added to dissolve compounds and plates were shaken for 10 min. Thawed cells in a single-cell suspension (10 000 cells in 20 µl per well) were dispensed using Multi-Drop Combi peristaltic dispenser (Thermo Scientific). Patient samples were suspended in Mononuclear Cell Medium (MCM; PromoCell), supplemented with 0.5 μg ml−1 gentamicin and 2.5 μg ml−1 amphotericin B, while cell lines were suspended in RPMI media (Lonza Bioscience), supplemented with 10% fetal bovine serum (FBS), 1% L-glutamine and 1% penicillin-streptomycin. Plates were then incubated for 72 h at 37 °C and 5% CO2. CellTiter-Glo 2.0 reagent (Promega) was used to measure cell viability according to the manufacturer’s instructions using a Pherastar FS plate reader. Luminescence measurements of cell viability were normalized to 100 mM benzethonium chloride-containing wells (positive control) and DMSO-only wells (negative control). Drug responses over the tested concentration range were quantified using the drug sensitivity score (DSS)(5).

***Drug combination testing***

BMNCs were thawed and suspended in StemSpan™ SFEM media (Stemcell technologies), plated in 5000 cell/well concentration on 384-well plates with dose-response matrices comprising 7 different concentrations for each drug as well as DMSO controls. For each single drug in combination, DSRT was performed as described above. SynergyFinder(6) web application was utilized to quantify drug combination synergy with the highest single agent (hsa) synergy score. A combinatorial synergistic activity was reported if the corresponding summary hsa synergy score was above 3, and antagonistic if less than -3, otherwise combination effect was considered as additive. A full list of hsa synergy and highest area synergy scores in Supplementary Table 8.

***CRISPR/Cas9 RUNX1 gene editing***

Baf3-*BCR-ABL1* were transfected with pU6-(BbsI) CBh-Cas9-T2A-mCherry (Addgene plasmid #64324) expressing CRISPR-Cas9 and sgRNA targeting mouse *runx1* gene exon 4 using Fugene HD transfection reagent (Promega) inducing transient expression of Cas9-sgRNA in the cells. After 72 hours, cells were sorted for GFP+ mCherry+ population using Sony SH800 cell sorter (Sony Biotechnology) into single cell/well in a round-bottom 96-well plate. Successfully grown clones were expanded and screened by capillary sequencing for detection of gene editing and successfully edited clone were selected and expanded. The predicted effect of the CRISPR-editing of *RUNX1* gene on protein structure was assessed using TIDE tool(3). The *RUNX1^-/mut^* cell line had an in-frame deletion (-3), predicted to have a deleterious effect on protein function, in addition to an out of frame (-1) deletion which was also validated by western blotting (Supplementary figure 9). The *RUNX1^wt/wt^* which has been transfected using the exact above-mentioned protocol, yet have the 2 wild type alleles of *RUNX1* gene with no mutations, was used as a control in addition to parental cell line that has not been transduced. *RUNX1* gene was cloned into LeGO-iC2 lentivirus expression vector (Addgene plasmid #27345) (transferred from pCMV5-AML1B plasmid, Addgene plasmid #12426) using ECORI-HF restriction enzyme (New England Biolabs), and lentivirally introduced in CRISPR-edited cell lines to induce re-expression of wild type gene into. Mutant *RUNX1^R162K^* was generated by applying mutagenesis kit (GeneArt Site-Directed Mutagenesis System, Invitrogen) to RUNX1-LeGO-iC2 plasmid and validated by capillary sequencing and lentivirally transduced in Baf3 and K562 cell lines. sgRNA targeting exon 2 of human *RUNX1* in lentiCRISPRv2 plasmid (Addgene #52961) was lentivirally introduced in K562 cell line. Details of all sgRNA, primers, oligos, plasmids and antibodies used in the experiments are in Supplementary Table 2.

***Generation of and phenotyping of CAR T cells***

CAR T cells were manufactured as previously described(7). Briefly, peripheral blood mononuclear cells (PBMCs) were collected using Ficoll-Paque Premium (GE Healthcare) density gradient centrifugation from buffy coats of healthy donors. The use of human material was approved by the Institutional review board of the Finnish Red Cross Blood Service. CD3/CD28 microbeads (Dynabeads Human T-Expander CD3/CD28, Life Technologies) at a 3:1 (microbead:T cell) ratio were employed to sort and activate T cells. For T cells culture, X-VIVO (Lonza) supplemented with 5% human AB-serum (Seralab) and 20 or 100 U/ml of IL-2 (Proleukin, Novartis) were used. Cell density was adjusted, by adding fresh culture medium, to 1×10^6^/ml on days 0-3 and 0.5×10^6^/ml on days 3-10. On day 2, T cells were transduced with 3^rd^ generation lentiviruses containing an IgG1-based CAR containing the extracellular single chain variable fragment and constant regions sequences of the CD19-targeting FMC63 antibody (AA 1-267, GenBank ID: HM852952.1), intracellular and transmembrane sequences of the co-stimulatory CD28 receptor, and the intracellular T lymphocyte activation domain of the TCR CD3ζ chain (a gift from Gianpietro Dotti, University of North Carolina, USA). T cells transduced using a vector lacking transgene (mock) were used as a negative control. T cells were phenotyped by flow cytometry using a panel of antibodies (Supplementary Table 3) and acquired on FACSAria II on days 10, 11, 15, and 22.

***Ex-vivo CART cytotoxicity assay***

CAR T-cell ex-vivo cytotoxicity assays were performed as previously described(8). In brief, frozen BMNCs were thawed and suspended in StemSpan™ SFEM media (Stemcell technologies). Cells were treated with DNase I (ThermoFisher Scientific) to remove clumps before counting. Cells were then seeded on a 96 flat-bottom well plate in 5x10^4^ cell/well in the presence of the indicated concentration of imatinib (1-10 000 nM) and/or the indicated ratio of CD19-CAR T- or mock-cells to blasts in a final volume of 100ul Stemspan media. The experiment was done in triplicates per each condition. The plate was incubated at 37 °C and 5% CO_2_. After 24 h, cell suspensions were moved into a V-bottom 96-well plate and washed by 100 µl of staining buffer (PBS + 2 mM EDTA + 0.5% BSA). After centrifugation (300 g for 5 min), Supernatant was discarded and the cells were resuspended in 25 μl staining buffer containing antibodies for CD34-FITC, CD4-PE-Cy7, CD3-APC, CD8-APC-H7 (all from BD Biosciences), CD19-Pacific Blue (clone SJ25-C1, Invitrogen), and CD69-BV510 (clone FN50, Sony Biotechnology) for 15 min at room temperature covered from light. Cells were then washed with 100 μl staining buffer and resuspended to 25 μl Annexin V binding buffer containing 0.5 µl AnnexinV-PE and 0.5 µl 7-AAD (both from BD Biosciences). Data were acquired using an iQue Screener Plus flow cytometer by sipping the entire volume of each well, thus measuring all cells, and analyzed using the ForeCyt software (edition 6.2, Intellicyt). Counts of viable cells in the CD34+ population per well were normalized to counts in DMSO-only wells (100% viability) and zero viable cells (0% viability).

***Variant analysis***

Analysis of DNA read data was mainly performed as previously described(9). Briefly, the Trimmomatic software(10) was used to pre-process sequence data and filtered paired-end reads were aligned to human reference genome build 38 (EnsEMBL v82) using BWA-MEM(11). Alignments were sorted using the SortSAM by coordinate, and the MarkDuplicate module of the Picard toolkit (Broad Institute) were used to mark PCR duplicates. Default parameters were used. Genome Analysis Toolkit (GATK) toolset(12) was then applied to identify variants and GATK resource files that had been converted from GRCh37 to GRCh38 using CrossMap(13) and chain files downloaded from EnsEMBL. For calling of variants, the GATK somatic short variant best practice (version 3.5) was employed, supplemented by estimation of the cross-sample contamination level and filtering of the 8-oxoguanine and deamination artifacts by several GATK4 tools (CalculateContamination, CollectSequencingArtifactMetrics, and FilterByOrientationBias). Variant calls were finally normalized using bcftools(14).

Variants were annotated and filtered using the Annovar tool(15) against the RefGene database. First, MuTect2 filters with a TLOD ≥ 6.3 or a TLOD ≥ 5.0 and supported by five or more independent COSMIC(16) were applied to all samples and variants other than those passing were filtered. For the trinucleotide profile, variant data were then filtered for false-positives by removing variants in intronic regions and with coverage ≤ 10, variant quality value ≤ 40, variant allele frequency ≤ 2.00%, strand odd ratio for SNVs ≥ 3.00, and strand odd ratio for indels ≤ 11.00, minor allele frequency ≥ 1% in the EPS and 1KG databases, minor allele frequency ≥ 1% in general, Finnish, and Non-European ExAC databases, and minor allele frequency ≥ 0.01% in general, Finnish, and Non-European ExAC databases and supported by less than six independent COSMIC samples. For functional analyses, the previous variant call set was filtered further by removing synonymous mutations and non-frameshift variants. Finally, mutations included in Fig. 3a were manually curated, missed known cancer variants checked and rescued, and variants inspected using Integrative Genomics Viewer 2.3.66 (Broad Institute). deconstructSigs(17) software was used to identify mutational signatures, using the with default parameters and cancer profiles downloaded from the COSMIC web site on September 2017. Structural rearrangements were called with Manta(18) from the same alignment files subjected to somatic short variant calling.

***MEME analysis and motif search***

Motif searches for breakpoints resolved using Manta were performed using the MEME algorithm(19). Specifically, the start and end coordinates of each deletion, inversion, duplication and insertion as well start coordinates of each translocation predicted by Manta was recorded and the surrounding 6, 10, 20, 50, 100 or 200 bp up- and downstream sequence region of the breakpoints extracted using Bedtools(20). Motifs enriched in the sequence set in comparison to 3^th^ order Markov chain constructed from the GRCh38 reference sequence were then searched using the MEME tool with the command-line parameters -dna, -revcomp, -mod zoops, -nmotifs 3, -markov_order 3 and -objfun classic.

***Analysis of RNA sequencing data***

Analysis of RNA-sequencing data was performed as previously described(9). Briefly, RNA-sequencing data pre-processing was done similar to WES data. Filtered paired-end reads were aligned using STAR(21) guided with EnsEMBL v82 gene models to human reference genome build 38 (EnsEMBL v82). Analysis was done using default settings (2-pass per-sample mapping), except for the overhang of the splice junctions that was set to 99. Aligned reads were sorted by coordinate using the SortSAM, PCR duplicates were identified by the MarkDuplicate module of the Picard toolkit, feature counts were generated using SubRead(22). Conversion of feature counts expression estimates was done using Trimmed Mean of M-values (TMM) normalisation(23). Genomic features with a CPM value ≤1.00 in less than half of samples were removed. Differential expression testing was then performed using the edgeR(24) software. In the statistical testing, comparisons between subject groups included factors for sequencing kit. The P‐values were adjusted with Storey’s Q-value for multiple comparisons(25). Differentially expressed genomic features were determined with Q ≤0.05 cutoff value. In data visualization and pathway analyses, we used batch-corrected CPM data for library preparation kit effect. Batches were corrected using the removeBatchEffect function in the package limma(26). Clustering of gene expression profiles was performed with both genes and samples using the Euclidean distance and Ward linkage method. RNA sequencing data of Lymphoid-BP samples from Branford S. et al, were similarly analyzed using limma and EdgeR softwares after mapping of total RNA reads with STAR. Read counts were then normalized among all patients and represented as CPM values. Differentially expressed genomic features were determined using p<0.001 as a cutoff value.

***Pathway enrichment analysis***

Pathway enrichment analysis was done using GSEA(27) software (Broad Institute) and Enrichr(28,29). In the GSEA analysis, a pre-ranked analysis was done using gene list prepared by ordering genes by their log-fold change between *RUNX1*^mut^ and *RUNX1*^wt^ groups in the batch-corrected CPM data. GSEA analysis was performed using default values. False discovery rate (FDR) q <0.05 was used as a threshold to filter analysis output. The Enrichr was applied to the protein coding genes significantly differentially expressed between *RUNX1*^mut^ and *RUNX1*^wt^ BP-CML patients groups, as well as *RUNX1^-/-^*, *RUNX1^-/mut^* and *RUNX1^wt/wt^* cell lines (3 biological replicates for each cell line setting).

**References**

1. Branford S, Wang P, Yeung DT, Thomson D, Purins A, Wadham C, et al. Integrative genomic analysis reveals cancer-associated mutations at diagnosis of CML in patients with high-risk disease. Blood. 2018 30;132(9):948–61.

2. Grossmann V, Kohlmann A, Zenger M, Schindela S, Eder C, Weissmann S, et al. A deep-sequencing study of chronic myeloid leukemia patients in blast crisis (BC-CML) detects mutations in 76.9% of cases. Leukemia. 2011 Mar;25(3):557–60.

3. Brinkman EK, Chen T, Amendola M, van Steensel B. Easy quantitative assessment of genome editing by sequence trace decomposition. Nucleic Acids Res. 2014 Dec 16;42(22):e168–e168.

4. Pemovska T, Kontro M, Yadav B, Edgren H, Eldfors S, Szwajda A, et al. Individualized systems medicine strategy to tailor treatments for patients with chemorefractory acute myeloid leukemia. Cancer Discov. 2013 Dec;3(12):1416–29.

5. Yadav B, Pemovska T, Szwajda A, Kulesskiy E, Kontro M, Karjalainen R, et al. Quantitative scoring of differential drug sensitivity for individually optimized anticancer therapies. Scientific Reports. 2014 Jun 5;4:5193.

6. Ianevski A, He L, Aittokallio T, Tang J. SynergyFinder: a web application for analyzing drug combination dose-response matrix data. Bioinformatics. 2017 Aug 1;33(15):2413–5.

7. Kaartinen T, Luostarinen A, Maliniemi P, Keto J, Arvas M, Belt H, et al. Low interleukin-2 concentration favors generation of early memory T cells over effector phenotypes during chimeric antigen receptor T-cell expansion. Cytotherapy. 2017;19(6):689–702.

8. Dufva O, Koski J, Maliniemi P, Ianevski A, Klievink J, Leitner J, et al. Integrated drug profiling and CRISPR screening identify essential pathways for CAR T-cell cytotoxicity. Blood. 2020 Feb 27;135(9):597–609.

9. Adnan Awad S, Kankainen M, Ojala T, Koskenvesa P, Eldfors S, Ghimire B, et al. Mutation accumulation in cancer genes relates to nonoptimal outcome in chronic myeloid leukemia. Blood Adv. 2020 Feb 11;4(3):546–59.

10. Bolger AM, Lohse M, Usadel B. Trimmomatic: a flexible trimmer for Illumina sequence data. Bioinformatics. 2014 Aug 1;30(15):2114–20.

11. Li H. Aligning sequence reads, clone sequences and assembly contigs with BWA-MEM. arXiv:13033997 [q-bio] [Internet]. 2013 Mar 16 [cited 2018 Jul 16]; Available from: http://arxiv.org/abs/1303.3997

12. McKenna A, Hanna M, Banks E, Sivachenko A, Cibulskis K, Kernytsky A, et al. The Genome Analysis Toolkit: A MapReduce framework for analyzing next-generation DNA sequencing data. Genome Res. 2010 Sep;20(9):1297–303.

13. Zhao H, Sun Z, Wang J, Huang H, Kocher J-P, Wang L. CrossMap: a versatile tool for coordinate conversion between genome assemblies. Bioinformatics. 2014 Apr 1;30(7):1006–7.

14. Li H. BFC: correcting Illumina sequencing errors. Bioinformatics. 2015 Sep 1;31(17):2885–7.

15. Wang K, Li M, Hakonarson H. ANNOVAR: functional annotation of genetic variants from high-throughput sequencing data. Nucleic Acids Res. 2010 Sep;38(16):e164.

16. Forbes SA, Beare D, Boutselakis H, Bamford S, Bindal N, Tate J, et al. COSMIC: somatic cancer genetics at high-resolution. Nucleic Acids Res. 2017 Jan 4;45(D1):D777–83.

17. Rosenthal R, McGranahan N, Herrero J, Taylor BS, Swanton C. deconstructSigs: delineating mutational processes in single tumors distinguishes DNA repair deficiencies and patterns of carcinoma evolution. Genome Biology. 2016 Feb 22;17:31.

18. Chen X, Schulz-Trieglaff O, Shaw R, Barnes B, Schlesinger F, Källberg M, et al. Manta: rapid detection of structural variants and indels for germline and cancer sequencing applications. Bioinformatics. 2016 15;32(8):1220–2.

19. Bailey TL, Elkan C. Fitting a mixture model by expectation maximization to discover motifs in biopolymers. Proc Int Conf Intell Syst Mol Biol. 1994;2:28–36.

20. Quinlan AR, Hall IM. BEDTools: a flexible suite of utilities for comparing genomic features. Bioinformatics. 2010 Mar 15;26(6):841–2.

21. Dobin A, Davis CA, Schlesinger F, Drenkow J, Zaleski C, Jha S, et al. STAR: ultrafast universal RNA-seq aligner. Bioinformatics. 2013 Jan 1;29(1):15–21.

22. Liao Y, Smyth GK, Shi W. The Subread aligner: fast, accurate and scalable read mapping by seed-and-vote. Nucleic Acids Res. 2013 May 1;41(10):e108.

23. Robinson MD, Oshlack A. A scaling normalization method for differential expression analysis of RNA-seq data. Genome Biology. 2010 Mar 2;11:R25.

24. Robinson MD, McCarthy DJ, Smyth GK. edgeR: a Bioconductor package for differential expression analysis of digital gene expression data. Bioinformatics. 2010 Jan 1;26(1):139–40.

25. Storey JD. A Direct Approach to False Discovery Rates. Journal of the Royal Statistical Society Series B (Statistical Methodology). 2002;64(3):479–98.

26. Ritchie ME, Phipson B, Wu D, Hu Y, Law CW, Shi W, et al. limma powers differential expression analyses for RNA-sequencing and microarray studies. Nucleic Acids Res. 2015 Apr 20;43(7):e47.

27. Subramanian A, Tamayo P, Mootha VK, Mukherjee S, Ebert BL, Gillette MA, et al. Gene set enrichment analysis: A knowledge-based approach for interpreting genome-wide expression profiles. PNAS. 2005 Oct 25;102(43):15545–50.

28. Kuleshov MV, Jones MR, Rouillard AD, Fernandez NF, Duan Q, Wang Z, et al. Enrichr: a comprehensive gene set enrichment analysis web server 2016 update. Nucleic Acids Res. 2016 Jul 8;44(W1):W90-97.

29. Chen EY, Tan CM, Kou Y, Duan Q, Wang Z, Meirelles GV, et al. Enrichr: interactive and collaborative HTML5 gene list enrichment analysis tool. BMC Bioinformatics. 2013 Apr 15;14:128.
